# Engineering High-Performance Li Metal Batteries through Dual-Gradient Porous Cu-CuZn Host

**DOI:** 10.1021/acsnano.4c00720

**Published:** 2024-05-16

**Authors:** Jianyu Chen, Guanyu Liu, Xuran Han, Hanbo Wu, Tao Hu, Yihang Huang, Shihao Zhang, Yizhou Wang, Zixiong Shi, Yu Zhang, Li Shi, Yanwen Ma, Husam N. Alshareef, Jin Zhao

**Affiliations:** † State Key Laboratory of Organic Electronics and Information Displays & Institute of Advanced Materials (IAM), Nanjing University of Posts & Telecommunications, 9 Wenyuan Road, Nanjing 210023, China; ‡ Materials Science and Engineering, King Abdullah University of Science and Technology (KAUST), Thuwal 23955-6900, Saudi Arabia; § New Energy Technology Engineering Lab of Jiangsu Province, School of Science, Nanjing University of Posts & Telecommunications, Nanjing 210023, China; ∥ Suzhou Vocational Institute of Industrial Technology, 1 Zhineng Avenue, Suzhou International Education Park, Suzhou 215104, China

**Keywords:** dual gradient, current collector, capillary action, molten Li, Li metal batteries

## Abstract

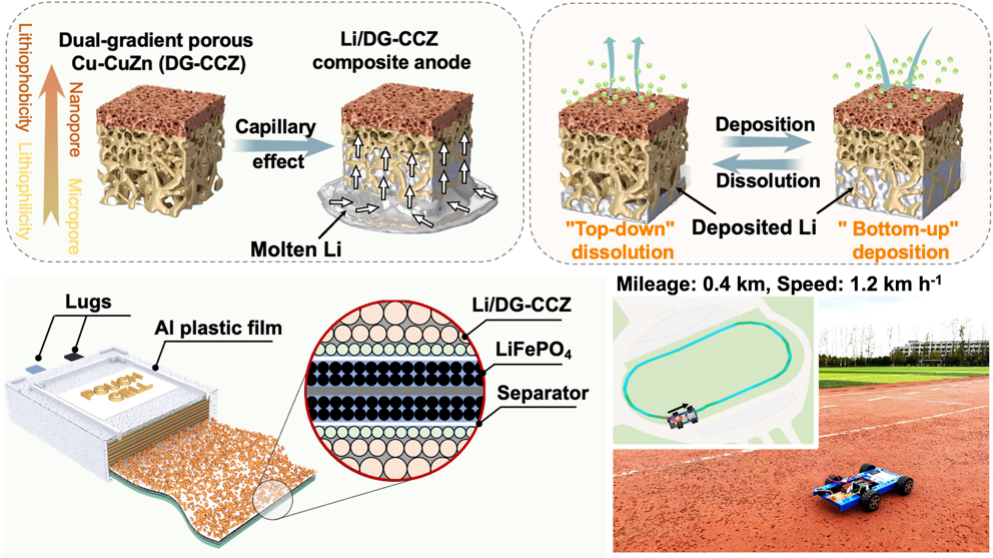

Porous copper (Cu) current collectors show promise in stabilizing Li metal anodes (LMAs). However, insufficient lithiophilicity of pure Cu and limited porosity in three-dimensional (3D) porous Cu structures led to an inefficient Li–Cu composite preparation and poor electrochemical performance of Li–Cu composite anodes. Herein, we propose a porous Cu-CuZn (DG-CCZ) host for Li composite anodes to tackle these issues. This architecture features a pore size distribution and lithiophilic-lithiophobic characteristics designed in a gradient distribution from the inside to the outside of the anode structure. This dual-gradient porous Cu-CuZn exhibits exceptional capillary wettability to molten Li and provides a high porosity of up to 66.05%. This design promotes preferential Li deposition in the interior of the porous structure during battery operation, effectively inhibiting Li dendrite formation. Consequently, all cell systems achieve significantly improved cycling stability, including Li half-cells, Li–Li symmetric cells, and Li-LFP full cells. When paired synergistically with the double-coated LiFePO_4_ cathode, the pouch cell configured with multiple electrodes demonstrates an impressive discharge capacity of 159.3 mAh g^–1^ at 1C. We believe this study can inspire the design of future 3D Li anodes with enhanced Li utilization efficiency and facilitate the development of future high-energy Li metal batteries.

Lithium (Li) metal anodes (LMAs) represent a promising energy storage technology based on electrochemical conversion reactions, offering a theoretical specific capacity of up to 3860 mAh g^–1^, surpassing conventional Li-ion intercalation chemistry.^1−4^ This surpasses the gravimetric capacity offered by conventional Li-ion intercalation chemistry, playing a pivotal role in advancing high-energy-density battery systems.^5−7^ However, the progress of LMAs faces two primary challenges: the nonuniform nucleation and deposition behavior of Li metal, as well as the subsequent growth of Li dendrites resulting from these processes.^8−12^ Researchers have extensively explored strategies to stabilize LMAs, encompassing anode structure design,^13−20^ anode interface engineering,^21−24^ separator engineering,^25−27^ and electrolyte modification.^28−34^ Through these efforts, the growth model of Li dendrites has been essentially elucidated. The suppression of Li dendrites and the development of techniques for inducing uniform nucleation/deposition of metallic Li have made significant advancements and garnered widespread consensus.^35−38^

A crucial breakthrough toward the practical application of Li metal batteries (LMBs) involves the integration of three-dimensional (3D) porous Cu current collectors (CCs), which have been proven to be effective in suppressing Li dendrite growth, thereby enhancing cycling stability.^39−43^ However, the challenge persists in two aspects. First, as an inactive unit in the battery structure, inactive Cu occupies space inefficiently, reducing the specific capacity of the composite anode and overall energy density. Increasing the porosity is imperative within porous Cu CCs to enhance the available space for Li loading. Presently, commercial Li-ion batteries employing graphite anodes achieve a capacity of 250.76 mAh g^–1^ at a thickness of 100 μm (90 μm graphite +10 μm Cu foil).^44−47^ To achieve similar capacity at the same thickness with a Li–Cu composite anode, a porosity of 50% becomes indispensable.^39^ Balancing heightened porosity demands while ensuring adequate conductivity, strength, ultrathin thickness, and appropriate pore size distribution (<10 μm) in porous Cu is essential.^39,48−52^ This guarantees stability during processing, welding, Li loading, and calendering in battery assembly processes. Composite anodes meeting these physical properties and processable characteristics have rarely been reported in the literature.

The challenge of scaling up the preparation of metallic Li with porous Cu while achieving uniform, dense, and high-loading Li within the current collector poses a significant obstacle.^53,54^ Previous strategies employed for fabricating composite Li metal anodes into current collectors, such as electrochemical plating, thermal melting infusion, and mechanical pressing, have shown limitations. Electrochemical deposition methods, though used extensively, proved laborious, expensive, and environmentally harmful at a large scale due to electrolyte waste.^55−58^ Therefore, pursuing efficient and scalable preparation techniques for Li/porous Cu composite electrodes is crucial in advancing Li metal batteries. Among various composite strategies, thermal molten Li methods have gained recognition for their advantages.^59−62^ Thermal infusion composite technology involving metallic Li enables denser Li deposition, enhancing volumetric energy density and reducing interfacial impedance.^63,64^ Moreover, this approach shows the advantages of simplicity and ease of control, as well as facilitates the efficient preparation of large-area Li composite anodes. The thermal infusion process entails heating Li to a molten state in a protective atmosphere and placing pretreated porous Cu CC in the molten Li, resulting in a composite electrode infused with metallic Li.^65,66^ Efforts to improve the wettability of foam metal involve incorporating lithiophilic species (e.g., Li-alloy metals, transition metal compounds, and heteroatom-doped carbon materials) and enhancing compatibility with molten Li.^67−70^ Introducing these lithiophilic species onto 3D porous Cu CCs can also help to attain uniform nucleation of Li,^71−75^ but the inhomogeneous ion concentration gradient within the porous framework could result in preferential Li deposition at the top and thus threaten the stable operation of the battery.^76−78^ Achieving a consistent “bottom-up” deposition mode and “top-down” dissolution mode is of utmost importance to overcome these issues. Nevertheless, the thermal infusion method for preparing Li composite anodes still faces two key challenges: insufficient wetting of lithiophobic porous Cu CCs by molten Li and inhomogeneous deposition behavior at the top area due to ion concentration gradients in single lithiophilic functional porous Cu.

Addressing these above-mentioned challenges necessitates synergistic improvements in molten Li wettability within porous Cu, rational electrode structure and composition design, as well as controlled diffusion of metallic Li. Regarding these aspects, an ideal porous Cu CC should possess the following characteristics: (1). A self-supporting structure with excellent structural stability and reversibility, which can ensure durability during the electrode fabrication process. (2). Well-designed lithiophilic gradients to introduce bottom-up deposition of metallic Li during molten Li infusion and electrochemical cycling process. (3). Rational pore structure gradient for rapid wetting of molten Li and internal infiltration and diffusion within the current collector. (4). Maintenance of uniform ion diffusion pathways even after Li dissolution. Therefore, designing innovative porous Cu current collectors that simultaneously satisfy these aspects is essential for the future development of Li–Cu composite anodes.

Herein, we present a porous Cu-CuZn current collector (CC) engineering for Li composite anodes, featuring a dual-gradient design exhibiting “lithiophilic to lithiophobic” and “microporous to nanoporous” characteristics. This dual-gradient porous Cu-CuZn enhances thermal molten Li metal wettability through its lithiophilicity gradient and maximizes capillary force via the pore structure gradient. This design enables efficient molten Li infusion during electrode preparation and targeted deposition-dissolution reactions of Li metal during battery cycling. Achieving a Coulombic efficiency exceeding 99.62% over 280 cycles in a Li/DG-CCZ_(melting)_ half-cell at 1 mA cm^–2^ and a cycling capacity of 1 mAh cm^–2^ demonstrates promising performance. Symmetric cells exhibit impressive electrochemical performance, maintaining a low polarization voltage of 25 mV for over 1000 h. The Li/DG-CCZ_(melting)_|LiFePO_4_|Li/DG-CCZ_(melting)_ electrode-based pouch cell demonstrates high electrochemical cycling stability, delivering a high specific discharge capacity of 159.9 mAh g^–1^, enduring over 100 cycles with high electrochemical cycling stability. When paired with the four-layered double-coated LiFePO_4_ cathode, the pouch cell with eight-layered Li/DG-CCZ_(melting)_ anodes can drive the prototype model vehicle that delivered a distance exceeding 0.4 km at a speed of at least 1.2 km h^–1^. We believe this work holds the promise of inspiring the design of efficient and scalable Li composite anodes and unlocking potential in high-energy Li metal battery development.

## Results and Discussion

### Design Concept of the DG-CCZ

**Figure 1:**
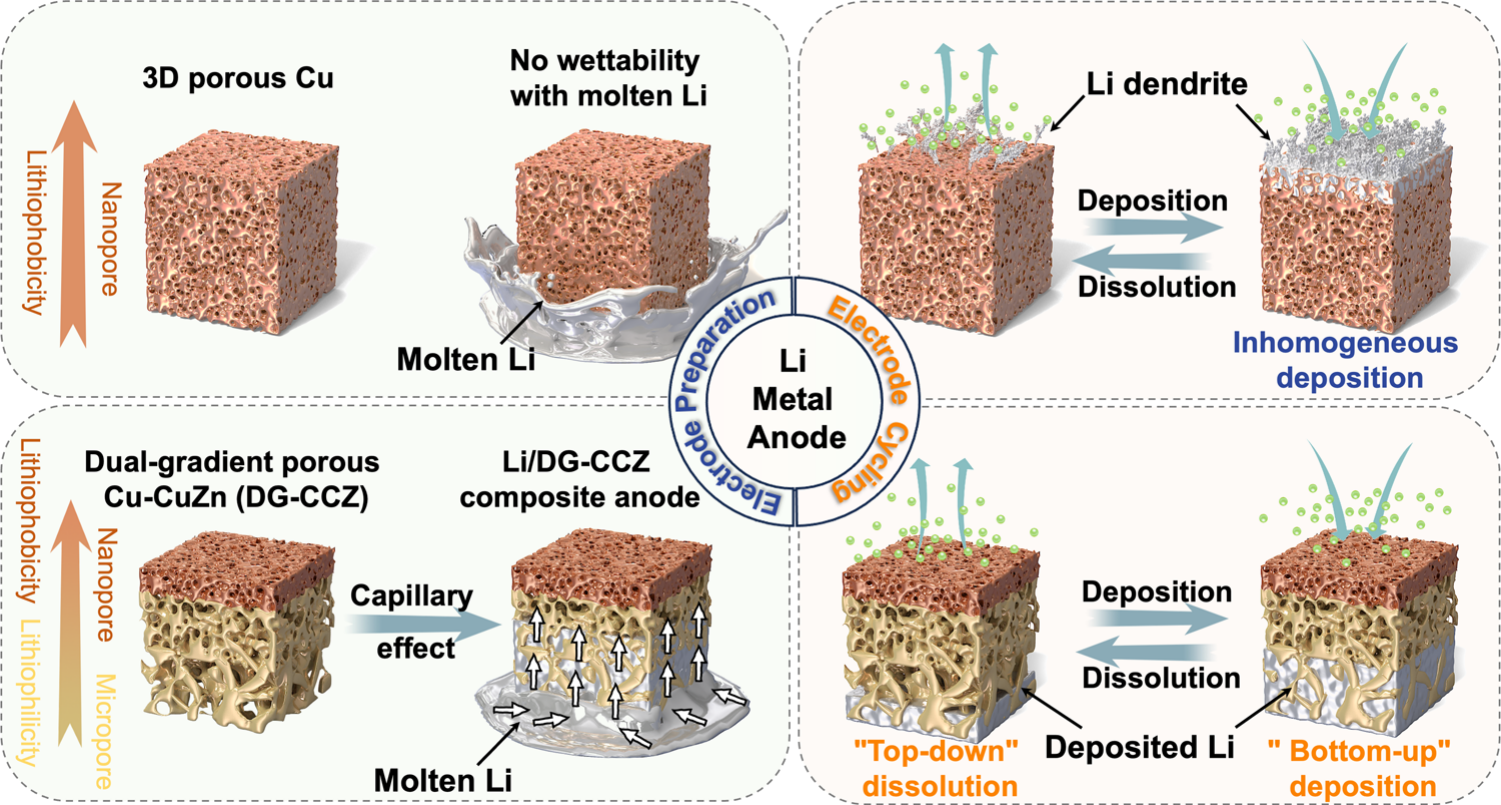
Schematic Illustration of electrode preparation and electrode cycling process. Schematic illustration of the nonwetting process of molten Li with 3D porous Cu (top left). Schematic illustration of Li plating/stripping process in 3D Cu anode (top right).Schematic illustration of capillary wetting of molten Li infusion in DG-CCZ current collector (bottom left). Schematic illustration of Li plating/stripping process in Li/DG-CCZ composite anode (bottom right).

For the preparation of the Li/DG-CCZ anode, which involves infusing molten Li into the 3D porous DG-CCZ structure, understanding the principles of liquid droplet wetting theory is crucial. The concept of wettability relies significantly on a droplet’s surface tension, as shown in Figure S1.^79,80^ A key factor in enhancing the wettability between Li and the porous Cu CCs involves reducing the surface tension of the molten Li. This reduction is pivotal in promoting effective interaction between Li and the porous substrate. Capillary forces play a critical role in governing the infiltration of molten Li within porous Cu CCs, beyond considerations of wettability. Thus, meticulous design of micro/nanoporous architectures consistently enhances capillary action, thereby facilitating the impregnation of molten Li. Moreover, the establishment of a lithiophilic-lithiophobic gradient emerges as a promising strategy for precisely depositing metallic Li within porous Cu current collectors. The assumed electrode preparation and electrode cycling process of the Li/DG-CCZ composite anode is schematically illustrated in Figure 1. The acquisition of a dual-functional gradient structure through powder sintering processes results in a distinctive bilayer architecture. This architecture exhibits a gradient distribution, transitioning from lithiophobicity to lithiophilicity along the electrode axis, while also featuring varying pore sizes from nanoscale to microscale across different regions. Throughout electrode fabrication, the advantages of this dual-gradient structure are harnessed to improve the capillary action of molten Li, enhancing its penetration velocity and depth within the Li/DG-CCZ matrix. In the electrode cycling process, a directed diffusion of metallic Li occurs within the DG-CCZ, characterized by “bottom-up” deposition and “top-down” dissolution. This controlled diffusion greatly contributes to efficient preparation and sustained cycling stability of the Li/DG-CCZ composite anode. Notably, this method shows promise for scalability, addressing persistent challenges in porous metal current collector design.

### Fabrication and Characterization of DG-CCZ Current Collector

**Figure 2:**
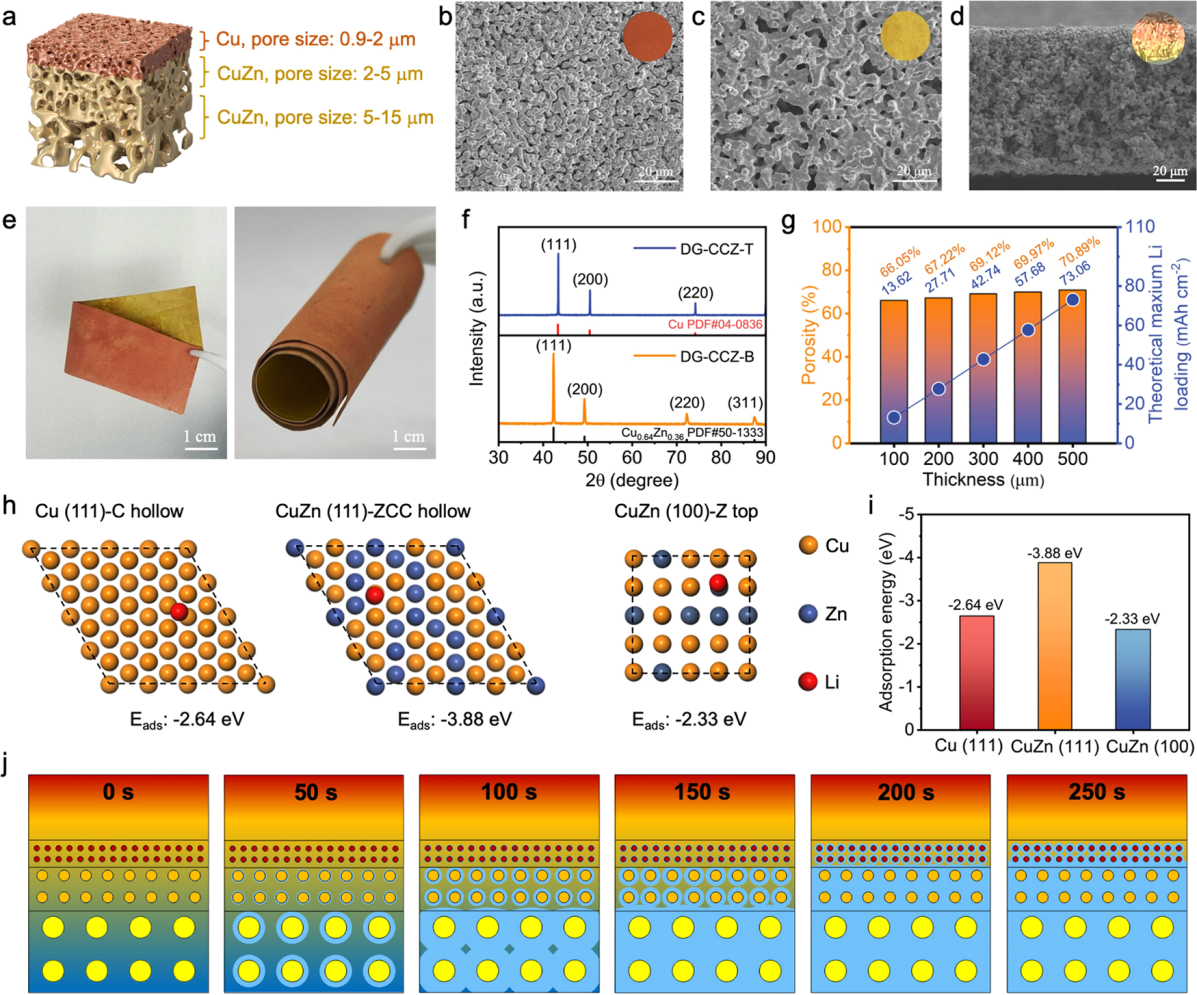
Morphological and structural characterizations of DG-CCZ current collectors. (a) Schematic diagram of pore size and composition distribution in DG-CCZ current collector. (b) Top-view, (c) bottom-view, and (d) cross-sectional-view SEM images of DG-CCZ current collector. (e) Photographs of the large-scaled DG-CCZ current collector with high flexibility. (f) XRD patterns of DG-CCZ current collector with top and bottom area. (g) Porosity of the DG-CCZ current collector with the thickness varied from 100 to 500 μm. (h) Adsorption sites of a Li atom on Cu(111), CuZn(111), and CuZn(100). (i) Adsorption energies between the Li atom and different substrates. (j) Finite element analysis simulation results of Li deposition behavior in DG-CCZ current collectors.

Integrated DG-CCZ CCs are prepared by powder sintering technology, as shown in Figures S2 and S3. Cu powders with different mesh sizes are used to construct a pore size gradient, and Zn powders were introduced to enable the lithophilicity difference.^81^ The pore size and composition are illustrated in Figure 2a, the DG-CCZ exhibits a three-layered structure with distinct compositions arranged from bottom to top. The lower and middle layers consist of CuZn, while the uppermost layer comprises pure Cu. Additionally, the pore size follows a sequential decrease from the bottom layer upward. The scanning electron microscopy (SEM) images in Figure 2b–d depict the top, bottom, and cross-sectional morphology of the DG-CCZ CC. The nanopatterned nature of the top region’s interconnected network structure and the microscale distribution of perforations in the bottom region are distinctly discernible from the SEM images. The digital images provided alongside the SEM images correspond to the color variation of the electrode surface in the top and bottom regions. Specifically, the brick-red color in the top region signifies the elemental presence of pure Cu, whereas the yellow color in the bottom region indicates the characteristic color of a CuZn alloy (Figures S4–S7). This observation clearly demonstrates the heterogeneous compositional distribution of the DG-CCZ CC along its vertical axis. Additionally, the cross-sectional SEM image of the DG-CCZ electrode exposes the contrasting porosity characteristics between the bottom and top regions. The bottom region reveals a porous and spongy structure, whereas the top region demonstrates a denser arrangement of pores, indicative of a longitudinal gradient in pore size within the electrode. The preparation of large-scale and highly flexible DG-CCZ electrodes can be realized through powder sintering technology, as illustrated in Figure 2e. As shown in Figure 2f, the X-ray diffraction (XRD) patterns exhibit the compositional constituents of the top (DG-CCZ-T) and bottom surface (DG-CCZ-B) materials of DG-CCZ CC, revealing a composition of pure Cu on the top surface and a Cu_0.64_Zn_0.36_ (donated as CuZn, PDF. No. 50–1333) alloy on the bottom surface. These findings unequivocally indicate distinct material compositional distributions along the longitudinal depth of the electrode.

A comprehensive exploration has been undertaken spanning sintering temperatures ranging from 350 to 550 °C (Figures S8 and S9). As shown in Figure S10, mercury porosimetry tests reveal that during the sintering process at 350 °C, both the Cu–Cu and Cu–Zn powder systems fail to achieve a complete reactive state, resulting in a porosity of 56.15%. Conversely, elevating the sintering temperature to 550 °C initiates a series of reactive processes between Cu and Zn powders, including diffusion, flow, dissolution, alloying, and homogenization reactions. Consequently, the porosity gradually decreases as pore closure commences (51.89%). At 450 °C, the DG-CCZ exhibits a balanced combination of both single-component sintering involving Cu powder and binary sintering involving Cu and Zn powders. This homogeneous sintering process results in a specific porosity value of 66.05%. Furthermore, it is noteworthy that the pore volume of the DG-CCZ (sintered at 450 °C) electrode exhibits a proportional increment with increasing electrode thickness. The porosity of DG-CCZ can be achieved through precise modifications in the design of presintering molds and the controlled adjustment of powder amounts during the slurry coating process. Precise manipulation of electrode pore volume offers an adaptable approach, allowing tailoring electrode designs to accommodate diverse demands concerning Li loading amount prerequisites (Figure 2g). The varying thicknesses of DG-CCZ (ranging from 100 to 500 μm) maintain an approximate porosity of ∼68.65%, offering substantial filling space for metallic Li. For example, the DG-CCZ with a thickness of 100 μm exhibits a high porosity of 66.05% can allow the filling of 3.986 mg of metallic Li, theoretically yielding an areal capacity of 13.62 mAh cm^–2^ and a gravimetric capacity of 403.51 mAh g^–1^. These values significantly surpass the areal and gravimetric capacities of 6.77 mAh cm^–2^ and 250.76 mAh g^–1^ provided by graphite composite anodes.^44^

To study the impact of these varying material compositions on lithiophilicity, a coalescence of XRD analysis data and density functional theory (DFT) analysis has been employed to estimate the variation in adsorption energy for Li atoms across different constituents. The optimized adsorption structures of individual Li atoms on the primary CuZn(111), CuZn(100), and Cu(111) crystallographic facets are shown in Figure 2h,i, with the resultant calculated binding energies summarized (Figures S11 and S12). Notably, based on the optimized calculations corrected by the Gibbs free energy (Δ*G*_ads_), the CuZn(111) electrode manifests a heightened binding energy of −3.88 eV, surpassing that of CuZn(100) (−2.33 eV) and Cu(111) (−2.64 eV), underscoring the robust interaction between Li atoms and the CuZn(111) surface.^82^ Evidently, the internal atomic sites within CuZn facilitate the adsorption of Li atoms, thereby designating Zn constituents within the CuZn electrode as lithiophilic sites. Consequently, these sites prompt the uniform nucleation of Li metal, which subsequently undergoes growth on the interior surface of the CuZn alloy electrode. Apart from the pronounced adsorption ability of lithiophilic Zn sites within the CuZn alloy, the high surface area created by the porous structure of the alloy plays a pivotal role in mitigating local current density and ensuring uniform charge distribution (Figure S13). This multifaceted function effectively circumvents the initiation of Li dendrites stemming from the disordered electron concentration gradient. According to the simulation results, as illustrated in Figure S13, the Li-ion charge distribution in the DG-CCZ electrode exhibits a smaller disparity, accompanied by a charge density of 0.08 A m^–2^ at the surface and 21.8 A m^–2^ at the bottom. Furthermore, lithiophilicity gradients with both small (denoted as LG-CCZ_(small pore)_) and large pore sizes (LG-CCZ_(big pore)_) demonstrate effectiveness in promoting a “bottom-up” deposition tendency (Figure S13). This finding underscores the importance of designing a lithiophilicity gradient. In contrast, the 3D Cu electrode demonstrates a more apparent disparity in Li-ion concentration, with a greater difference between the top and bottom regions. This substantial variance in Li-ion concentration distribution is poised to induce a preferential deposition of metallic Li at the electrode’s top area, coupled with a gradual deposition at the bottom portion. For the DG-CCZ electrode, the diminished difference in Li-ion concentration distribution is anticipated to mitigate the dissimilarity in deposition between its top and bottom regions. Furthermore, the current density distribution of the DG-CCZ electrode exhibits a more distinct contrast than that of the 3D Cu electrode, as evidenced by Figure S13. The current density at the top of the 3D Cu electrode surpasses that of the bottom region in the electrode. Compared to the 3D Cu electrode, the DG-CCZ electrode’s current density distribution reveals a trend wherein the top region displays lower values than the middle and bottom regions. Additionally, Figures 2j and S14 illustrate the simulated process of Li metal deposition, including electrochemical plating and thermal infusion on the DG-CCZ electrode. Li metal deposition manifests as a bottom-to-top growth process within the DG-CCZ electrode. Conversely, for the pore size gradient Cu (PG-Cu) (Figure S15) electrode, due to the inherent lithiophobic properties of Cu and the shorter diffusion distance from the top electrode to Li ions, metallic Li exhibits a tendency to nucleate at the top of the porous electrode. These results strongly support that the DG-CCZ CC promotes Li metal’s “bottom-up” deposition model due to its gradient-distributed lithiophilic active sites along the thickness direction, thus enabling homogeneous Li metal deposition.

### Fabrication of Li/DG-CCZ Composite Anode and Investigation of Li Plating/Stripping Behavior

**Figure 3:**
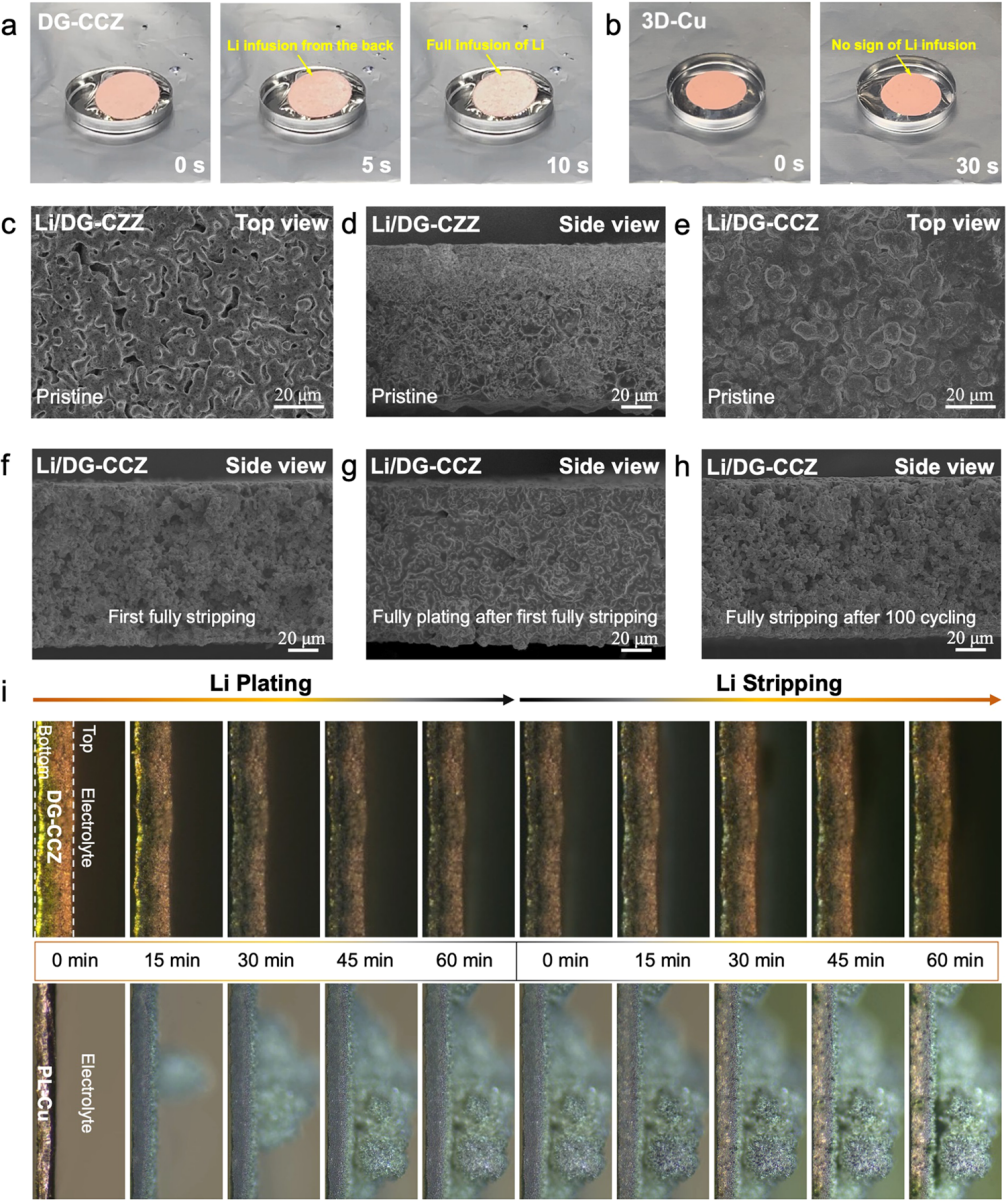
Capillary wettability behavior of molten Li and Li plating/stripping behavior in DG-CCZ current collectors. (a) Dynamic wetting process of molten Li in DG-CCZ, (b) dynamic wetting process of molten Li in a lithiophobic 3D Cu structure, (c) top-view SEM image of Li/DG-CCZ after molten Li wetting, (d) cross-sectional SEM image of Li/DG-CCZ after molten Li wetting, revealing internal material structures, (e) bottom-view SEM image of Li/DG-CCZ after molten Li wetting, providing insights into wetting depth, (f) cross-sectional SEM image of Li/DG-CCZ_(melting)_ after electrochemical dissolution following molten Li wetting, illustrating the effects of electrochemical dissolution on the DG-CCZ CC, (g) cross-sectional SEM image of Li/DG-CCZ_(melting)_ after electrochemical deposition of metallic Li following Li dissolution, showcasing the process of Li dissolution and deposition, (h) cross-sectional SEM image of DG-CCZ current collector after repeated cycles of Li deposition and dissolution, highlighting potential changes in the current collector’s morphology, (i) dynamic electrochemical behavior of metallic Li deposition and dissolution in DG-CCZ (top) and PL-Cu (bottom) captured by in situ optical electron microscopy.

Next, the infusion of molten Li into different 3D Cu CCs is tested. Figure 3a illustrates the molten Li infusion process wherein DG-CCZ CCs are meticulously infused with the molten Li. Upon contact between the DG-CCZ and molten Li, rapid diffusion of the molten Li ensues within the DG-CCZ structure, facilitated by two pivotal functionalities of the DG-CCZ electrode. First, the lithiophilic gradient at the bottom area of the electrode enhances the wettability of molten Li, while second, the bottom-up pore gradient intensifies the capillary action of molten Li, thereby augmenting the diffusion rate of molten Li within the pore network. In contrast, the conventional lithiophobic 3D Cu electrode, when immersed in molten Li, fails to adsorb molten metallic Li into its porous structure due to the inherent lithiophobic nature of Cu (Figure 3b). The unaltered surface tension of molten Li arising from this lithiophobicity impedes the elevation of metallic Li’s wettability. Consequently, the capillary diffusion of molten Li is thwarted, thereby curtailing the effective utilization of capillary action to facilitate its dispersion characteristics. To further verify the wetting behavior and capillary action of molten Li within DG-CCZ, an inverted DG-CCZ electrode was immersed in molten Li to investigate the wetting phenomenon. It is evident that even after prolonged contact between the electrode and molten Li for 30 s (Figure S16), the molten Li fails to infiltrate the electrode structure. This observation effectively establishes the constructive role of DG-CCZ ’s structural design in facilitating the directed diffusion of molten Li.

Then, the stripping/plating behaviors of Li on the DG-CCZ electrode are explored. After removing the DG-CCZ electrode from molten Li, the morphology characterization of the Li-absorbed composite anode (Li/DG-CCZ) is shown in Figure 3c–e. It is evident that the electrode surface retains a porous structure that remains unfilled, while the bottom portion is uniformly enveloped by metallic Li (Figure 3e). Furthermore, even after charging, the DG-CCZ electrode retains a substantial amount of porous architecture (Figure 3f). Performing subsequent Li deposition experiments on the delithiated blank electrode demonstrated the uniform filling of metallic Li within the electrode’s porous structure, devoid of dendritic growth. The SEM image of this electrode reveals the homogeneous electroplating of metallic Li within the DG-CCZ structure (Figures 3g and S17). Subsequent dissolution of this electrode after 100 cycles confirmed the structural integrity (Figure 3h). To further verify the reversibility of the Li/DG-CCZ_(melting)_ during electrochemical cycling, an in situ optical cell was employed to visualize the dynamic deposition and dissolution behaviors of Li. As shown in Figure S18, Li metal exhibits a top-down dissolution trend from the molten-infused electrode and maintains exceptionally high electrochemical reversibility during subsequent redeposition and redissolution processes. It indicates that the Li/DG-CCZ_(melting)_ composite anode plays a beneficial role in facilitating the dissolution/deposition behavior of Li metal. Figure 3i presents the dynamic evolution of Li in the blank DG-CCZ electrode; the corresponding capacity is 10 mAh cm^–2^, which is close to the maximum Li loading value. It can be seen that the “silver-gray” Li was deposited from the DG-CCZ CC in a “bottom-up” manner and subsequently stripped in a “top-down” fashion, indicating that the DG-CCZ shows a directly inducing function for uniform Li plating and stripping. Conversely, the conventional planar Cu CC displays a disordered dendritic growth. These observations align coherently with the outcomes garnered from the SEM images (Figures S19 and S20). These experimental findings lead to the following conclusions: (1). Introducing lithiophilic sites within the porous Cu CC is a prerequisite for achieving molten Li thermal infusion. (2). The rate and depth of molten Li infiltration within porous structures are closely related to capillary forces. (3). Introducing a dual gradient of lithiophilic-lithiophobic regions and micro-nano pores alters the local current density on internal and external surfaces of the CCs, influencing both the “bottom-up” deposition and “top-down” dissolution of metallic Li.

### Electrochemical Performance Evaluation

**Figure 4:**
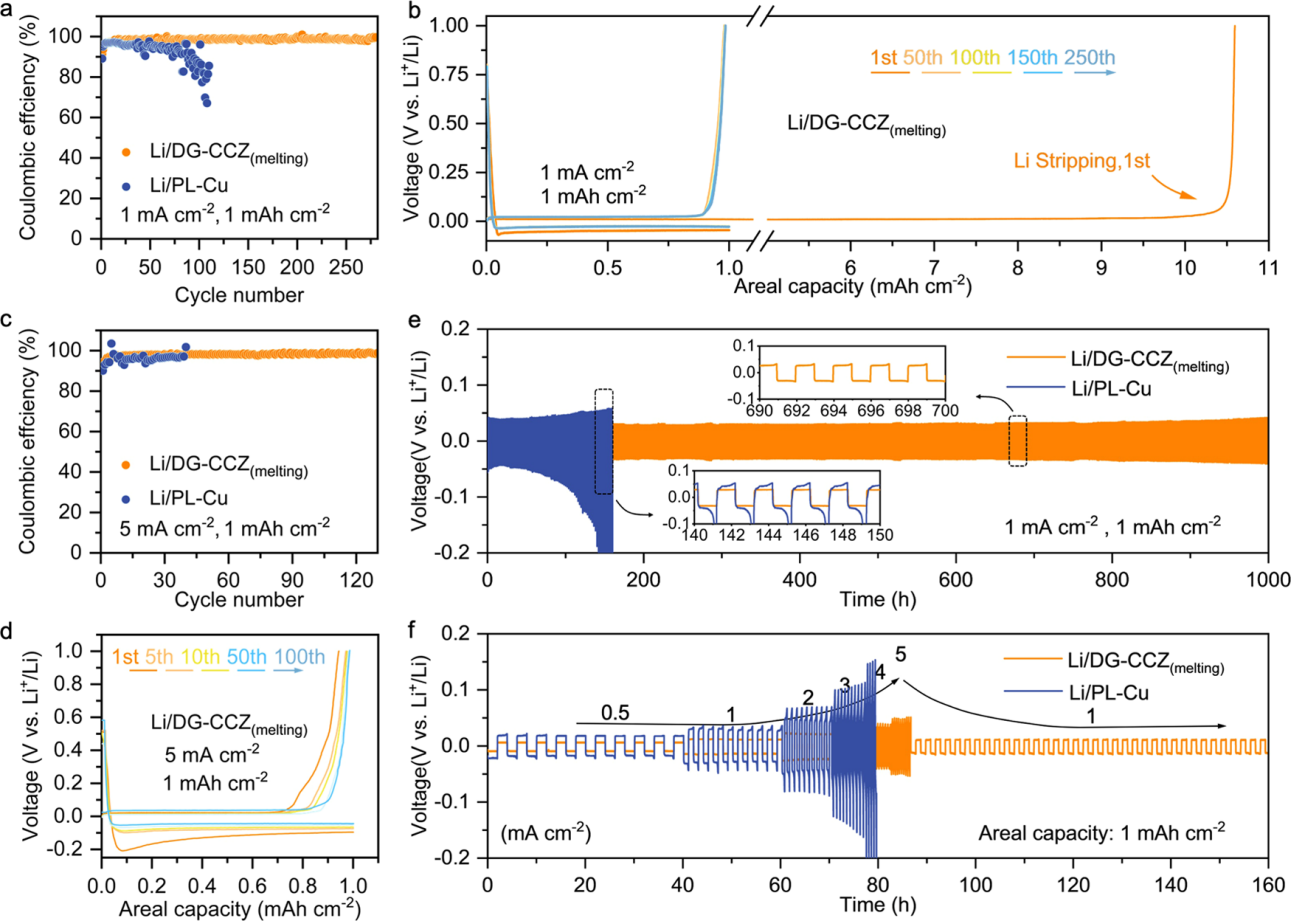
Electrochemical stability evaluation of DG-CCZ current collectors. (a) Coulombic efficiency plots of integrated Li/DG-CCZ_(melting)_ composite electrode. (b) Charge–discharge voltage profiles of integrated Li/DG-CCZ_(melting)_ composite electrode. (c) Coulombic efficiency plots of integrated Li/DG-CCZ_(melting)_ composite electrode under the current density of 5 mA cm^–2^ and cyclic capacity of 1 mAh cm^–2^. (d) Charge–discharge voltage profiles of Li/DG-CCZ_(melting)_ current collectors under 5 mA cm^–2^ current density and cyclic capacity of 1 mAh cm^–2^. (e) Voltage–time profiles of Li/DG-CCZ_(melting)_ composite electrode symmetric cells at the current density of 1 mA cm^–2^ and cyclic capacity of 1 mAh cm^–2^. (f) Voltage–time profiles of Li/DG-CCZ_(melting)_ composite electrode symmetric cells under various current densities and cyclic capacity of 1 mAh cm^–2^.

To reveal the electrochemical performance of the Li/DG-CCZ_(melting)_ composite anodes, a series of tests are conducted (Figure 4). First, a charging test was conducted to assemble the uniformly Li-filled composite anode in a half-cell, employing a metallic Li foil as the counter electrode (Figure S21). Upon reaching a cutoff voltage of 1 V, the representative Li/DG-CCZ_(melting)_ composite anode can deliver an areal capacity of 10.59 mAh cm^–2^, corresponding to a gravimetric capacity of 320.91 mAh g^–1^, as shown in Figure 4b. These values are similar to the theoretical predictions and significantly surpass the standard capacity of graphite composite anodes (250.76 mAh g^–1^, 6.77 mAh cm^–2^),^44^ exhibiting the potential application in full batteries to create high energy densities. Quantified Li deposition and dissolution cycles were conducted upon the complete dissolution of metallic Li from the electrode. Figure 4a shows the enhanced Coulombic efficiency of the Li/DG-CCZ_(melting)_ electrode with an average value of 98.55% for 280 cycles compared to the Li/PL-Cu electrode. To further verify the cycling stability of the composite anode prepared by molten Li under high-rate conditions, the Li/DG-CCZ_(melting)_, compared to the Li/PL-Cu electrode, still exhibited superior cycling stability under a higher current density of 5 mA cm^–2^. To eliminate the influence of prefilled Li on subsequent Li nucleation, a blank DG-CCZ CC was directly assembled in a half-cell to assess its Coulombic efficiency, as illustrated in Figure S22. DG-CCZ showcased a high Coulombic efficiency of 93.85% in the initial cycle, with a nucleation overpotential of 25 mV. This starkly contrasts the nucleation overpotential (65 mV) observed on the PL-Cu CC (Figure S23). Under an increased current density of 5 mA cm^–2^, the DG-CCZ CC maintained a high Coulombic efficiency of ∼98.79% for over 100 cycles (Figure 4c). In contrast, the Coulombic efficiency of the PL-Cu CC exhibited rapid and substantial fluctuations, underscoring DG-CCZ’s capacity to sustain electrochemical reversibility and stability even under elevated current densities (Figure S24). Evaluation of high reversible capacity plating/stripping of metallic Li in half-cell systems is another key criterion in addition to high current density testing to evaluate the efficacy of porous hosts. Figure S25 illustrates that DG-CCZ exhibits extended cycling performance with highly overlapped charge–discharge curves, indicating the inherently excellent structural and electrochemical stability of DG-CCZ. Symmetric cell configurations were established by utilizing Li/DG-CCZ_(melting)_ composite electrodes to assess the electrochemical stability (Figure 4e). Notably, the extended cycling stability and minimal hysteresis (∼25 mV) over 1000 h were observed for Li/DG-CCZ_(melting)_ electrodes at a current density of 1 mA cm^–2^, accompanied by a cycling capacity of 1 mAh cm^–2^. Conversely, Li/PL-Cu electrodes experienced substantial voltage fluctuations within a mere 6 h of cycling. To study the influence of the Li/DG-CCZ_(melting)_ structure on Li plating/stripping behavior, electrochemical impedance spectra (EIS) were tested under conditions of 1 mA cm^–2^ and 1 mAh cm^–2^ (Figure S26 and Table S1). Compared to the PL-Cu electrode, the DG-CCZ electrode exhibits higher interface stability and lower transfer charge resistance (Table S1). This observation signifies the advantageous deposition-dissolution kinetic behavior of metallic Li on the surface of the DG-CCZ electrode. Figure 4f exhibits the rate performance of the initially prepared Li/Cu electrodes at varying current densities ranging from 0.5 to 5.0 mA cm^–2^ while maintaining a constant cycling capacity of 1 mAh cm^–2^. Notably, the Li/DG-CCZ_(melting)_ electrode showed consistent voltage hysteresis (ranging from 6 to 52 mV), which proved to be notably lower than that exhibited by the Li/PL-Cu electrode (ranging from 19 to 154 mV). Additionally, symmetric cells can also be established by Li/DG-CCZ_(plating)_ composite electrodes with precisely controlled Li loading. As shown in Figure S27, Li/DG-CCZ_(plating)_ electrodes exhibited a low voltage polarization of ∼12 mV for over 1400 h at 1 mA cm^–2^ and 1 mAh cm^–2^, even when subjected to a current density of 10 mA cm^–2^ and a consistent areal capacity of 1 mAh cm^–2^, Li/DG-CCZ_(plating)_ demonstrated superior cycling stability with lower hysteresis (40 mV) compared to symmetric cells featuring Li/PL-Cu electrodes (Figure S28). A more comprehensive evaluation of the anode structure’s stability involves Li metal deposition/dissolution tests under high-capacity cycling conditions. As shown in Figures S29 and S30, it becomes evident that DG-CCZ exhibits exceptional cycling stability with a consistent voltage fluctuation during Li plating and stripping processes. In contrast, the Li plating and stripping behavior on PL-Cu demonstrated irregular voltage fluctuations due to the unstable interface resulting from disordered Li deposition on the planar Cu surface. The enhanced electrochemical performance of DG-CCZ CCs can be attributed to their substantial surface area, which facilitates reduced local current density and ensures a consistent electrolyte flux for efficient Li-ion transportation. Moreover, the appropriately sized pores induce uniform Li deposition while inhibiting dendrite growth, and the ample space accommodates the significant volume changes inherent to Li metal.

**Figure 5:**
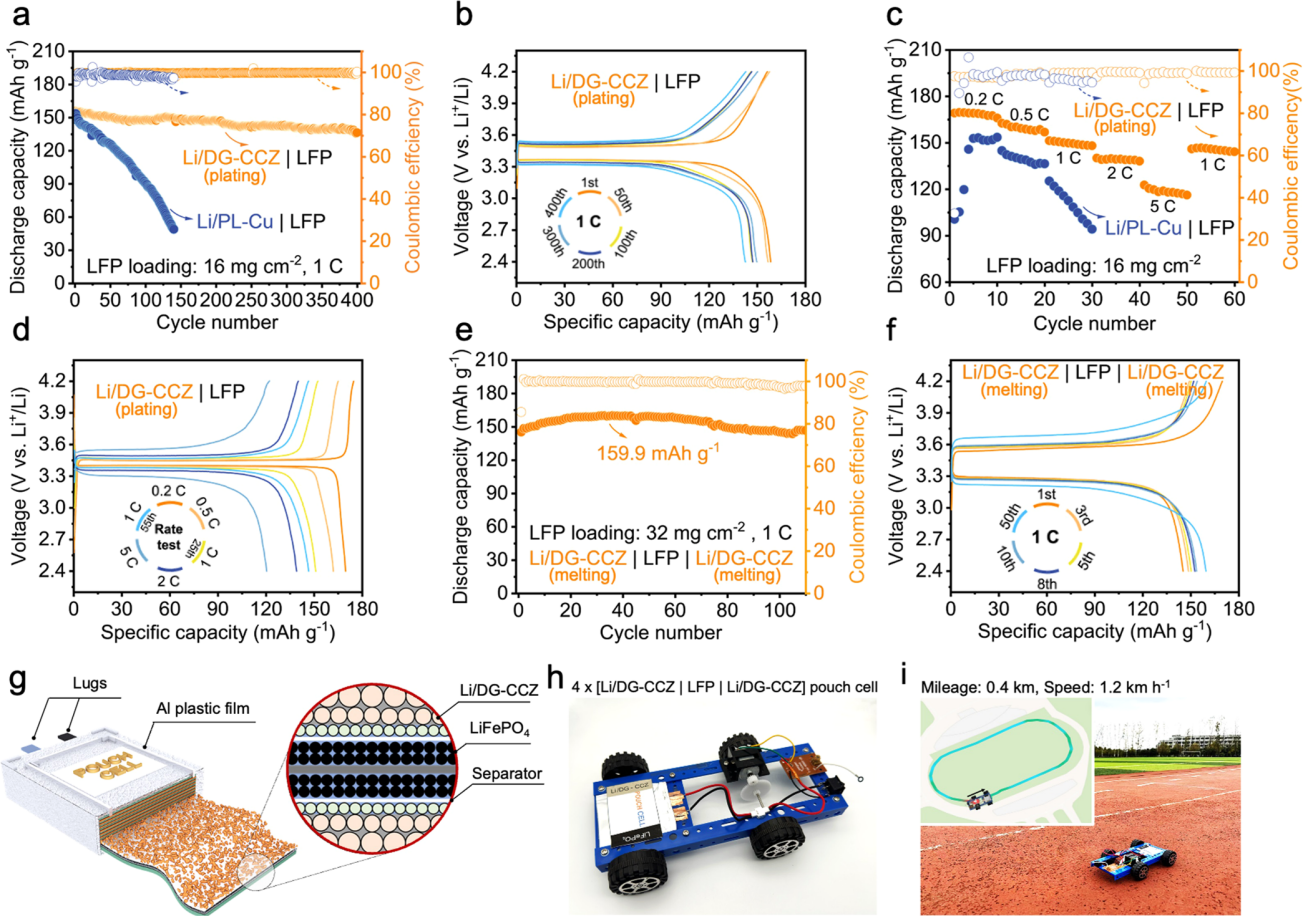
Comprehensive electrochemical performance of LFP cathode-based full cells. (a) Long-term electrochemical performance of Li/DG-CCZ_(plating)_|LFP and Li/PL-Cu | LFP full cells at 1C. (b) Charge–discharge voltage profiles of Li/DG-CCZ_(plating)_|LFP full cell at 1C. (c) Long-term electrochemical performance of Li/DG-CCZ_(plating)_|LFP and Li/PL-Cu|LFP full cells under different rates. (d) Charge–discharge voltage profiles of Li/DG-CCZ_(plating)_|LFP full cell under different rates. (e) Long-term electrochemical performance of Li/DG-CCZ_(melting)_|LFP|Li/DG-CCZ_(melting)_ pouch cell. (f) Charge–discharge voltage profiles of Li/DG-CCZ_(melting)_|LFP|Li/DG-CCZ_(melting)_ pouch cell. (g) Schematic illustration of the structure of Li/DG-CCZ_(melting)_|LFP|Li/DG-CCZ_(melting)_ pouch cell. (h) Digital photographs of a prototype model vehicle powered by an individual pouch cell, consisting of four double-sided coated cathodes and eight-layered anodes. (i) Digital photographs of the movement trajectory diagram by a prototype model vehicle powered by the as-prepared pouch cell.

To explore the practical applicability of Li/DG-CCZ_(melting)_ anodes, comprehensive evaluations were conducted using LiFePO_4_ (LFP) full cells. The ensuing long-term cycling performance is presented in Figure S31. It is evident that LFP full cells incorporating Li/DG-CCZ_(melting)_ exhibit distinct charge–discharge profiles during the initial discharge while demonstrating comparable capacities. Upon undergoing an extensive 280-cycle test at 1C, the Li/DG-CCZ_(melting)_|LFP full cell exhibits a high 91.21% improvement in capacity retention over the Li/PL-Cu|LFP cell. The excellent cycling stability observed in the Li/DG-CCZ_(melting)_|LFP full cell underscores the inherent structural robustness of Li/DG-CCZ_(melting)_, contributing to an extended cycle life. Additionally, the electrochemical deposition strategy allows for precise control over the Li loading amount to match different cathodes with specific capacities, thereby enhancing the utilization efficiency of Li metal. Therefore, we employed the electrochemical deposited Li with DG-CCZ current collector (Li/DG-CCZ_(plating)_) to construct full batteries with an N/P ratio of 2:1. As shown in Figure 5a,b, the Li/DG-CCZ_(plating)_|LFP full cell also exhibited a high discharge specific capacity of 155.6 mAh g^–1^ and extended cycle life after 400 cycles. In contrast, the Li/PL-Cu|LFP full cell exhibited poor cycling performance with low capacity retention (Figures 5b and S32). Furthermore, the rate capability of the Li/DG-CCZ_(plating)_|LFP full cell significantly outperforms that of the Li/PL-Cu|LFP full cell (Figures 5c,d and S33). At corresponding current densities of 0.2, 0.5, 1, and 2 C, the Li/DG-CCZ_(plating)_|LFP cell achieves discharge capacities of 168.50, 159.47, 149.63, and 139.17 mAh g^–1^, respectively. Impressively, even under the demanding current density of 5 C, the Li/DG-CCZ_(plating)_|LFP cell maintains a notable discharge capacity of 118.64 mAh g^–1^, in stark contrast to the Li/PL-Cu|LFP cell’s capacity of only 16.51 mAh g^–1^. The comparative analysis underscores the superior cyclic performance of the Li/DG-CCZ_(plating)_|LFP cell compared to its Li/PL-Cu counterpart, notably regarding discharge capacity, Coulombic efficiency, and capacity retention. The as-prepared DG-CCZ CC, with its heightened porosity and structural stability, led well to the fabrication of large-scale pouch cells. Moreover, benefiting from the simplicity and efficiency of the molten infusion method for electrode fabrication, Li/DG-CCZ_(melting)_ electrodes have been directly employed as the anode for pouch batteries. As shown in Figure S34 and Table S2, the pouch cell comprises double Li/DG-CCZ_(melting)_ anodes and double-coated LFP cathodes encapsulating the separator, sealed within an Al-plastic package. The maximum discharge capacity of the Li/DG-CCZ_(melting)_|LFP|Li/DG-CCZ_(melting)_ pouch cell measures 159.9 mAh g^–1^ at 1C (Figure 5e). Throughout the long-term cycling, the pouch cell exhibits substantial capacity retention of 91.74%. The charge–discharge curves of Li/DG-CCZ_(melting)_|LFP|Li/DG-CCZ_(melting)_ display a distinct overlap and a stable plateau, characterized by a low voltage hysteresis of approximately 0.23 V (Figure 5f). To validate the feasibility of assembling a multielectrode stacked pouch cell configuration, we utilized eight-layered Li/DG-CCZ_(melting)_ composite anodes and four-layered double-sided LFP cathodes to construct a pouch cell, as depicted in Figure 5g. Notably, the excellent flexibility of DG-CCZ empowers the pouch cell to effectively power the timer across diverse bending states (Figure S35), underscoring its practical viability across various scenarios and deformation conditions. To enhance the practical performance of such large-area flexible electrodes, the pouch cell, comprising multiple layers of cathodes and anodes, was assembled to evaluate its electrochemical properties (Figure S36 and Table S3). Notably, the pouch cell assembled with eight anodes and four double-side coated cathodes demonstrated a high discharge capacity of 159.3 mAh g^–1^ (Figure S37 and Table S4) and a high gravimetric energy density of 165.7 Wh kg^–1^ based on the total mass of the cathodes and anodes (Figure S38), highlighting its potential for practical applications. For practical application demonstration, a prototype model vehicle is fabricated, whose power source is a pouch cell consisting of four double-sided coated cathodes and eight Li/DG-CCZ_(melting)_ anodes (Figure 5h). Using the Li/DG-CCZ_(melting)_ anode-based pouch cell, the prototype model vehicle delivered a distance exceeding 0.4 km at a speed of at least 1.2 km h^–1^, affirming the viability and efficiency of the multilayered pouch cell configuration (Figure 5i). This substantial enhancement highlights the promising prospects of employing Li/DG-CCZ_(melting)_ as the anode solution in future Li-ion battery industries.

## Conclusions

This study introduces a dual-gradient porous Cu current collector engineering that exhibits distinctive lithiophilicity-lithiophobicity gradients and varying pore sizes from micrometers to nanometers. Innovatively, the dual-gradient engineering design allows targeted Li metal wetting during electrode preparation and coordinated diffusion within the gradient porous Cu during cycling, ensuring controlled deposition and dissolution. With a high porosity of 66.05%, the DG-CCZ electrode yields a high specific capacity of up to 320.91 mAh g^–1^ integrated with molten Li metal. Li/DG-CCZ anodes in half-cells and symmetric cells exhibit enhanced cycling. Notably, the pouch cell with the Li/DG-CCZ_(melting)_|LiFePO_4_|Li/DG-CCZ_(melting)_ electrode shows impressive electrochemical cycling stability and a discharge capacity of 159.9 mAh g^–1^. These findings emphasize Li/DG-CCZ ’s potential as a graphite substitute in Li-ion batteries, leading to a noteworthy enhancement in energy density. Successful Li/DG-CCZ integration promises enhanced Li-ion battery performance, enabling higher energy density and efficient energy storage systems. These findings also highlight the potential for efficient and scalable Li composite anodes, offer an inspiring opportunity to stimulate future innovations and explore frontiers in battery technology development.

## Experimental Section

### Materials

Spherical Cu powders (particle size category: 10, 5 and 1 μm; purity ≥99.99%) were purchased from Suzhan Smart Tech Co., Ltd. Spherical Zn powders (particle size category: 10 and 5 μm; purity ≥99.99%) were purchased from Suzhan Smart Tech Co., Ltd. Lithium iron phosphate (LFP), poly(vinylidene fluoride) (PVDF) powder, *N*-methylpyrrolidone (NMP), and Super P were purchased from Suzhou DuoDuo Chemical Tech Co. All chemicals can be used directly without further purification.

### Fabrication of DG-CCZ Current Collector

A theoretical simulation-based approach was employed to fabricate a DG-CCZ current collector, utilizing Cu and Zn powders of varying particle sizes as the building blocks. The schematic representation of the powder sintering process is illustrated in Figures S2 and S3. The lower layer (A) of the DG-CCZ current collector was fabricated by mixing Cu and Zn powders with a particle size of 10 μm and poly(vinylidene fluoride) (PVDF) powder in a ratio of 80:20:0.1, respectively. *N*-methylpyrrolidone served as the solvent. The mixture was spread onto a quartz plate using a doctor blade, followed by vacuum drying at 80 °C to obtain the CuZn-10 μm stacked layer A. Similarly, the intermediate layer (B) was prepared by mixing Cu and Zn powders with a particle size of 5 μm and PVDF powder in the same ratio of 80:20:0.1 and then spread on layer A. The upper layer (C) was formed by compacting a mixture of Cu powder with a particle size of 1 μm and PVDF powder. The stacking sequence included layer A with a thickness of 50 μm, layer B with 40 μm, and layer C with 20 μm, resulting in a final electrode thickness of 110 μm. Subsequently, another quartz plate was placed naturally on the stacked ABC electrodes without applying additional pressure. The stacked sample was sintered in a tubular furnace under a H_2_–Ar mixed atmosphere, with a sintering temperature of 450 °C, and a heating rate of 10 °C min^–1^, for 2 h. Subsequently, the naturally cooled sample was washed with ethanol and deionized water to obtain a DG-CCZ current collector with a thickness of ∼100 μm.

### Fabrication of Li/DG-CCZ Composite Anode

1. Thermal melting method. The infiltration experiments were conducted within an argon gas atmosphere maintained within a glovebox to prevent oxidation of the materials. The metal Li was heated to specific temperatures of 300 °C. A meticulously crafted iron tweezer was employed to remove the surface oxide layers from the molten Li, ensuring a clean and reactive surface. Subsequently, the DG-CCZ current collector was immersed in the molten Li to observe the diffusion and distribution behavior of the molten Li within the porous structure.
2. Electrochemical plating method. The cells were assembled using a standard CR2032 coin-type cell. A Celagard 2400 porous polypropylene with a diameter of 19 mm is selected as the separator for all cells. The electrolyte solution with the amount of 60 μL is used for all cells composed of 1 M bis (trifluoromethane) sulfonamide lithium (LiTFSI) salt in a mixed solvent of 1,2-dimethoxyethane (DME) and 1,3-dioxolane (DOL) with 1:1 volume ratio containing LiNO_3_ (1 wt %). All of the cells were assembled in a glovebox (an argon environment) with water and oxygen content less than 0.1 ppm.

### Instruments and Characterizations

Morphology observation was conducted with a Hitachi S-4800 field-emission SEM. The transfer process of Li-containing samples (after electrochemical plating or stripping treatment) was protected under the Ar atmosphere. Porosity analysis was tested by a mercury porosimeter (MicroActive AutoPore V 9600) with pressure from 0.1 to 60,000 psi. XRD patterns were recorded on a Bruker D8 Avance diffractometer equipped with a Cu Kα radiation source. In situ optical microscopy (YUESCOPE, YM710TR) was conducted to capture the dynamic Li plating/stripping behavior.

### Electrochemical Measurements

The specific surface area, pore size distribution, and porosity of the current collector were determined using mercury porosimetry (MicroActive AutoPore V 9600). Morphology observation was conducted with a Hitachi S-4800 field-emission scanning electron microscope (SEM) The transfer process of Li-containing samples (after electrochemical plating or stripping treatment) was protected under the Ar atmosphere. X-ray diffraction patterns were recorded using X-ray diffractometer equipment with Cu Kα radiation (λ = 0.15418 nm) operating at 30 kV (XRD; Bruker, D2 Avance).

## Supporting Information Available

All cell test details; schematic illustration of capillary force at different substrates; schematic diagram of the powder sintering preparation process of DG-CCZ current collectors; model diagram of the powder sintering process; photograph of as-fabricated DG-CCZ current collectors; photographs and SEM images of DG-CCZ sintering at different temperatures; porosity analysis for DG-CCZ current collectors; adsorption energy (DFT) of a Li atom on different substrates and COMSOL simulation results of different current collectors; dynamic wetting process of molten Li on DG-CCZ, SEM images of Li/DG-CCZ after Li plating and stripping; charge–discharge voltage profiles and Coulombic efficiencies of PL-Cu and DG-CCZ current collectors; electrochemical impedance spectroscopy plots of DG-CCZ and PL-Cu half-cells, voltage-time profiles of Li/DG-CCZ and Li/PL-Cu, charge–discharge voltage profiles of Li/DG-CCZ_(melting)_|LFP and Li/PL-Cu|LFP full cell; optical microscope images of pouch cells; schematic diagram of pouch cell configuration and charge–discharge voltage profiles of Li/DG-CCZ_(melting)_|LFP|Li/DG-CCZ_(melting)_ pouch cell; electrochemical impedance fitted parameters, electrochemical performance, and theoretical calculation of the maximum areal capacity density of Li in DG-CCZ; physical property data of DG-CCZ current collectors; and comparison of different Cu-based current collectors and electrochemical performance (PDF)
