# Supplementary material for: Engineering High-Performance Li Metal Batteries through Dual-Gradient Porous Cu-CuZn Host
Source: ACS Nano. 2024 May 16;18(21):13662–74. doi: 10.1021/acsnano.4c00720 (PMC11140834; doi:10.1021/acsnano.4c00720)
Supplement: Supplementary file 1 — nn4c00720_si_001.pdf [file nn4c00720_si_001.pdf]

Supporting Information

Engineering High-Performance Li Metal Batteries through Dual-Gradient Porous Cu-CuZn Host

Jianyu Chen, 1 Guanyu Liu, 1 Xuran Han, 1 Hanbo Wu, 1 Tao Hu, 1 Yihang Huang, 1 Shihao Zhang, 1 Yizhou Wang, 1,2 Zixiong Shi, 2 Yu Zhang, 3 Li Shi, 1 Yanwen Ma, 1,4 Husam N. Alshareef 2,* and Jin Zhao 1,**

1 State Key Laboratory of Organic Electronics and Information Displays & Institute of Advanced Materials (IAM), Nanjing University of Posts & Telecommunications, 9 Wenyuan Road, Nanjing 210023, China.

2 Materials Science and Engineering, King Abdullah University of Science and Technology (KAUST), Thuwal 23955-6900, Saudi Arabia.

3 New Energy Technology Engineering Lab of Jiangsu Province, School of Science, Nanjing University of Posts & Telecommunications, Nanjing, 210023, China.

4 Suzhou Vocational Institute of Industrial Technology, 1 Zhineng Avenue, Suzhou International Education Park, Suzhou 215104, China.

Corresponding Author

*Yanwen Ma - E-mail: iamywma@njupt.edu.cn

*Husam N. Alshareef - E-mail: husam.alshareef@kaust.edu.sa

*Jin Zhao - E-mail: iamjzhao@njupt.edu.cn

EXPERIMENTAL SECTION

Materials. Spherical Cu powders (particle size category: 10 μm, 5 μm and 1 μm; purity ⩾99.99%) were purchased from Suzhan Smart Tech Co., Ltd. Spherical Zn powders (particle size category: 10 μm and 5 μm; purity ⩾99.99%) were purchased from Suzhan Smart Tech Co., Ltd. Lithium iron phosphate (LFP), polyvinylidene fluoride (PVDF) powder, N-methyl pyrrolidone (NMP), and Super P were purchased from Suzhou DuoDuo Chemical Tech Co. All chemicals can be used directly without further purification.

Fabrication of DG-CCZ current collector. A theoretical simulation-based approach was employed to fabricate a DG-CCZ current collector, utilizing Cu and Zn powders of varying particle sizes as the building blocks. The schematic representation of the powder sintering process is illustrated in Figure S2 and S3. The lower layer (A) of the DG-CCZ current collector was fabricated by mixing Cu and Zn powders with a particle size of 10 μm and polyvinylidene fluoride (PVDF) powder in

a ratio of 80:20:0.1, respectively. N-methylpyrrolidone served as the solvent. The mixture was spread onto a quartz plate using a doctor blade, followed by vacuum drying at 80 ˚C to obtain the CuZn-10 μm stacked layer A. Similarly, the intermediate layer (B) was prepared by mixing Cu and Zn powders with a particle size of 5 μm and PVDF powder in the same ratio of 80:20:0.1 and then spread on layer A. The upper layer (C) was formed by compacting a mixture of Cu powder with a particle size of 1 μm and PVDF powder. The stacking sequence included layer A with a thickness of 50 μm, layer B with 40 μm, and layer C with 20 μm, resulting in a final electrode thickness of 110 μm. Subsequently, another quartz plate was placed naturally on the stacked ABC electrodes without applying additional pressure. The stacked sample was sintered in a tubular furnace under a H_2-Ar mixed atmosphere, with a sintering temperature of 450 ˚C, a heating rate of 10 ˚C/min, for 2 hours. Subsequently, the naturally cooled sample was washed with ethanol and deionized water to obtain a DG-CCZ current collector with a thickness of ~ 100 μm.

Fabrication of Li/DG-CCZ composite anode.

1. Thermal melting method. The infiltration experiments were conducted within an argon gas atmosphere maintained within a glovebox to prevent oxidation of the materials. The metal Li was heated to specific temperatures of 300 ˚C. A meticulously crafted iron tweezer was employed to remove the surface oxide layers from the molten Li, ensuring a clean and reactive surface.

Subsequently, the DG-CCZ current collector was immersed in the molten Li to observe the diffusion and distribution behavior of the molten Li within the porous structure.

2. Electrochemical plating method. The cells were assembled using a standard CR2032 coin-type cell. A Celagard 2400 porous polypropylene with a diameter of 19 mm is selected as the separator for all cells. The electrolyte solution with the amount of 60 μL is used for all cells composed of 1 M bis (trifluoromethane) sulfonamide lithium (LiTFSI) salt in a mixed solvent of 1, 2-dimethoxyethane (DME) and 1, 3-dioxolane (DOL) with 1:1 volume ratio containing $LiNO_3$ (1 wt%). All the cells were assembled in a glove box (an argon environment) with water and oxygen content less than 0.1 ppm.

Instruments and Characterizations. Morphology observation was conducted with a Hitachi S-4800 field-emission SEM. The transfer process of Li-containing samples (after electrochemical plating or stripping treatment) was protected under the Ar atmosphere. Porosity analysis was tested by a mercury porosimeter (MicroActive AutoPore V 9600) with pressure from 0.1 to 60,000 psi. XRD patterns were recorded on a Bruker D8 Avance diffractometer equipped with a Cu-Ka radiation source. In-situ optical microscopy (YUESCOPE, YM710TR) was conducted to capture the dynamic Li plating/stripping behavior.

Electrochemical Measurements. The specific surface area, pore size distribution, and porosity of the current collector were determined using mercury porosimetry (MicroActive AutoPore V 9600

). Morphology observation was conducted with a Hitachi S-4800 field-emission scanning electron microscope (SEM) The transfer process of Li-containing samples (after electrochemical plating or stripping treatment) was protected under the Ar atmosphere. X-ray diffraction patterns were recorded using X-ray diffractometer equipment with Cu Kα radiation (λ=0.15418 nm) operating at 30 kV (XRD; Bruker, D2 Avance).

Half-Cell Tests. The molten Li-infused electrode and blank electrode were assembled to test the Coulomb efficiency and electrochemical plating/stripping behavior, respectively. For molten Li-infused electrodes (Li/DG-CCZ), the half-cells are charged to 1 V to ensure the complete dissolution of Li within the composite anode, allowing for the calculation of the corresponding areal and gravimetric capacities. Subsequent repeated plating/stripping tests are conducted to determine the Coulombic efficiency. The PL-Cu current collector is similarly deposited with 10.59 mAh cm^{-2}, followed by charging to dissolve Li, and then repeated charging and discharging cycling are performed to test Coulombic efficiency. For the half-cell tests on blank electrodes, DG-CCZ or PL-Cu current collector was used as the working electrode, and Li foil was used as the counter electrode. The cell was activated between 0.1 and 1 V before electrochemical plating and stripping. The fixed amount of Li metal was deposited on the working electrode and stripped out up to 1 V under the specific current density conditions.

Symmetrical-Cell Tests. The molten Li-infused electrode and electrochemical-plated electrode were assembled in the symmetrical cell to test cycling stability, respectively. For molten Li-infused electrodes, the Li/DG-$CCZ_{(melting)}$ electrodes were directly assembled in the symmetrical cell under a specific experimental condition. Li/PL-Cu (~10.59 mAh cm^{-2}) electrodes were also assembled in the symmetrical cell for comparison. For the electrochemical-plated electrode, 2 mAh cm^{-2} of Li was deposited on a DG-CCZ or PL-Cu blank current collector at a current density of 1 mA cm^{-2} and then reassembled at the same electrode in the cell. The cells were cycled at a specific experimental condition.

$LiFePO_4$ Full-Cell Tests. To prepare a standardized cathode, Lithium iron phosphate ($LiFePO_4$) powder, conductive carbon black, and polyvinylidene difluoride (PVDF) were mixed at a mass ratio of 8:1:1 in N-methylpyrrolidone (NMP) solution to form a slurry after vigorous stirring. The as-prepared slurry was pasted on the carbon-coated aluminum foil, dried in an air oven for 0.5 h, and then transferred to a vacuum-drying oven for 12 h. The prepared $LiFePO_4$ electrode was cut into circular disks with a diameter of 12 mm. The loading mass of $LiFePO_4$ is about 32 mg cm^{-2}. In the assembly of full batteries, composite anodes obtained through molten Li infusion and those obtained via electrochemical deposition were respectively utilized as anodes. For the former, the molten Li-infused anodes were directly integrated into full batteries for charge-discharge testing, evaluating their discharge capacity, Coulombic efficiency, and lifespan. The molten Li-infused

anodes can deliver a gravimetric capacity of ~10.59 mAh cm^{-2} (Figure 4b) and the corresponding N/P ratio is ~3.85:1. For the latter, to construct a full cell with a low N/P ratio, 5.5 mAh cm^{-2} of Li metal was predeposited in the DG-CCZ current collector, and the resulting Li/DG-CCZ served as the anode. The corresponding negative versus positive (N/P) ratio is 2:1. The voltage window for the full battery test is 2.5-4.2 V.

Li/DG-CCZ$_{(melting)}$ | LFP | Li/DG-CCZ$_{(melting)}$ Pouch-Cell Tests. The pouch cell contains a single Li/DG-CCZ$_{(melting)}$ anode and double-coated LFP and then is sealed by Al plastic films. The specific parameters of all pouch cells are summarized in Table S2 and S3.

Li/DG-CCZ$_{(melting)}$ | LFP | Li/DG-Cu$_{(melting)}$ Pouch Cell Tests: The pouch cell was fabricated by sandwiching one Li/DG-CCZ$_{(melting)}$ anode between two LFP cathodes and then sealed by Al plastic films. To satisfy the operation of the battery, the capacity of the DG-CCZ anode (molten Li-infused electrode, ~10.59 mAh cm^{-2}) is slightly higher than that of the cathode ($LiFePO_4$, 32 mg cm^{-2}, ~5.5 mAh cm^{-2}). The device based on the homemade pouch cell was assembled in our laboratory to achieve a practical Li metal battery. The dimensions of the pouch cell are (0.03 × 0.03 × 0.002) m^3.

The Finite Element Analysis (FEA) Simulation Details. In this study, Finite Element Analysis (FEA) was conducted using COMSOL software based on the electrochemical model previously described.[1] We constructed a 3D geometric model tailored to the specifics of the battery system

and electrode configuration. The model facilitates the computation of current density, Li-ion concentration distribution, and the Li plating process on current collectors, employing Hook's law, the Butler-Volmer equation, and the Nernst-Planck equation, respectively. To enhance computational efficiency, the geometric model was simplified, reflecting the essential features of the actual electrode structure.

The 3D simulation utilized the tertiary current distribution module for transient analysis. The electrolyte's initial Li-ion concentration was set to 1 M, with the model's upper surface designated as Li foil to serve as the counter electrode, supplying Li^+. An external potential of 50 mV vs. Li/Li^+ was applied. The simulation incorporated 3D Cu electrodes, accounting for variations in pore size distribution and hole dimensions. A separation between electrodes was established to emulate the diaphragm's thickness. To accurately portray lithiophilicity differences, the 3D Cu electrode was segmented into two regions, each characterized by distinct diffusion coefficients and exchange current densities, with an external potential of -50 mV vs. Li/Li^+ applied. The lithiophobic electrode featured a lithium-ion diffusion coefficient of 1×10^{-12} m^2 s^{-1} and a reference exchange current density of 5 mA cm^{-2}. Conversely, the lithiophilic electrode's diffusion coefficient was 7×10^{-9} m^2 s^{-1}, with a reference exchange current density of 100 mA cm^{-2}. In models lacking a lithiophilicity gradient, a uniform electrode diffusion coefficient of 5×10^{-12} m^2 s^{-1} and an exchange current density of 80 mA cm^{-2} were used. To mimic experimental conditions,

a local current density of 15 mA cm^{-2} was applied at the working electrode's base. The simulation results presented derive from data captured at 60 seconds into the simulation.

The 2D simulation, employing both the 2D phase field and tertiary current distribution modules, offers transient analysis similar to its 3D counterpart. This simulation's geometry mirrors that of the 3D model, with Li foil as the counter electrode at the top. Various 3D Cu electrodes, defined at the bottom, adhere to parameters established in the 3D simulation. Data for this section were extracted from transient simulations at intervals of 0, 50, 100, 150, 200, and 250 s.

Finite element simulations based on COMSOL were conducted to investigate the infiltration process of molten Li into granular electrodes in two dimensions, utilizing both laminar flow and level set interfaces in fluid dynamics. Various granular electrode models were simulated, considering differences in particle size, packing arrangement, and surface wettability. Specifically, the laminar flow computations were based on the Shallow Channel Approximation. The initial condition was set with an initial liquid level at the bottom of the simulation domain, while the top and bottom boundaries were designated as open boundaries, and the left and right boundaries were set as no-slip wall boundaries. The model domain was discretized using a free triangular mesh, with boundary layers imposed on particle surfaces and boundaries to ensure the accuracy of the liquid-level results. The transient solver employed for a solution was PARADISO, an inbuilt solver in the software, with acceleration facilitated by the Anderson accelerator for rapid convergence. The time-varying infiltration process of molten Li into granular electrode interiors under different models was obtained through simulation.

Theoretical Calculation of the Density Functional Theory. All DFT calculations were performed using the plane-wave density function as implemented by Quantum Espresso.[2] The Generalized gradient approximation in conjunction with Perdew-Burke-Ernzerhof functional

(PBE-GGA) was used to describe the electron exchange-correlation interaction.[3] The DFT-D3 method of Grimme was used to describe long-range Van der Waals (vdW) interactions.[4] In structural relaxation, the energy cutoff for the plane-wave basis expansion and convergence threshold for the force on each relaxed atom was set to 60 Ry and 0.01 eV $Å^{-1}$, respectively. The CuZn alloy (111) and (100) facet was constructed with at least four metal atomic layers, being thick enough for energy convergence. A 3×3×1 Monkhorst-Pack k-point set-up was used for geometry optimization of (111) and (100) facets, respectively geometry optimization, and a vacuum slab of 15 Å was used for surface isolation to prevent interaction between two layers. For the determination of the free energy (ΔG_{ads}) between Li and facet, the following equation was utilized: $\Delta G_{ads} = G_{Li\,+\,(111/100)\,facet} - G_{(111/100)\,facet} - G_{Li}$, where: the $G_{Li\,+\,(111/100)\,facet}$, $G_{(111/100)\,facet}$ and G_{Li} represents the free energy of the total energy of the system, the facet, and the isolated Li atom, respectively. The free energy is calculated as $G = E_{DFT} + E_{ZPE} + \int C_p dT - TS$, where the E_{DFT} is the electronic energy calculated by DFT, E_{ZPE} is the zero-point energy (ZPE), C_{p} is heat capacity, T and S is temperature and entropy, respectively. The E_{ZPE}, TS and $\int C_P dT$ for each reaction intermediates can be calculated by the following equations, respectively:

$$E_{ZPE} = \frac{1}{2}\sum_i h v_i$$

$$-TS = k_B T \sum_i \ln\left(1 - e^{-\frac{h v_i}{k_B T}}\right) - \sum_i h v_i \left(\frac{1}{e^{\frac{h v_i}{k_B T}} - 1}\right)$$

$$\int C_P dT = \sum_i h v_i \left(\frac{1}{e^{\frac{h v_i}{k_B T}} - 1}\right)$$

In the equations above, h, v and k_B are Planck constant, vibrational frequencies, and Boltzmann constant, respectively. Besides, only when we calculate the free energy of reaction intermediates, the zero-point energy, entropy, and enthalpy are needed to calculate.

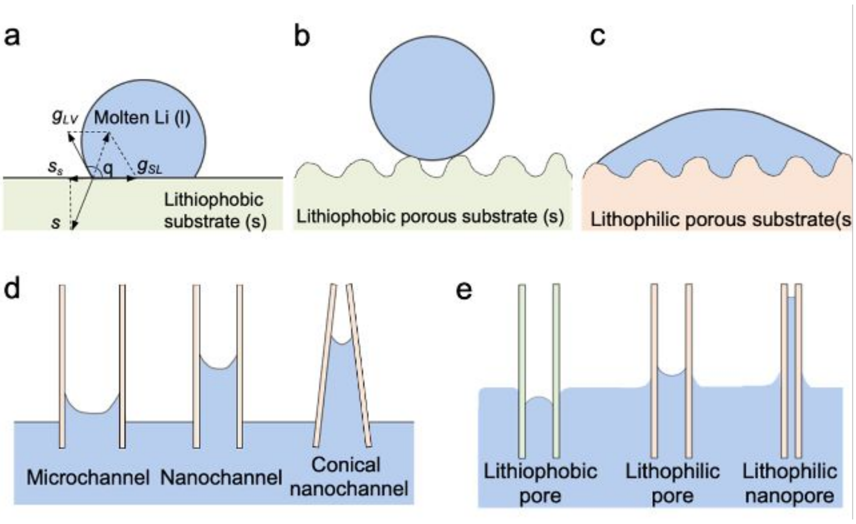

Figure S1. (a) Vectorial equilibrium for a molten Li droplet resting on a solid lithiophobic surface to balance three forces. (b) The static state of a molten Li droplet resting on a porous lithiophobic surface. (c) The static state of a molten Li droplet resting on a porous lithopholic surface. (d) Schematic representation of capillary force at the same lithophilic channels with different scales. (e) Schematic representation of capillary force at different channels with lithiophobic pore, lithophilic pore, and lithiophilic nanopore structure.

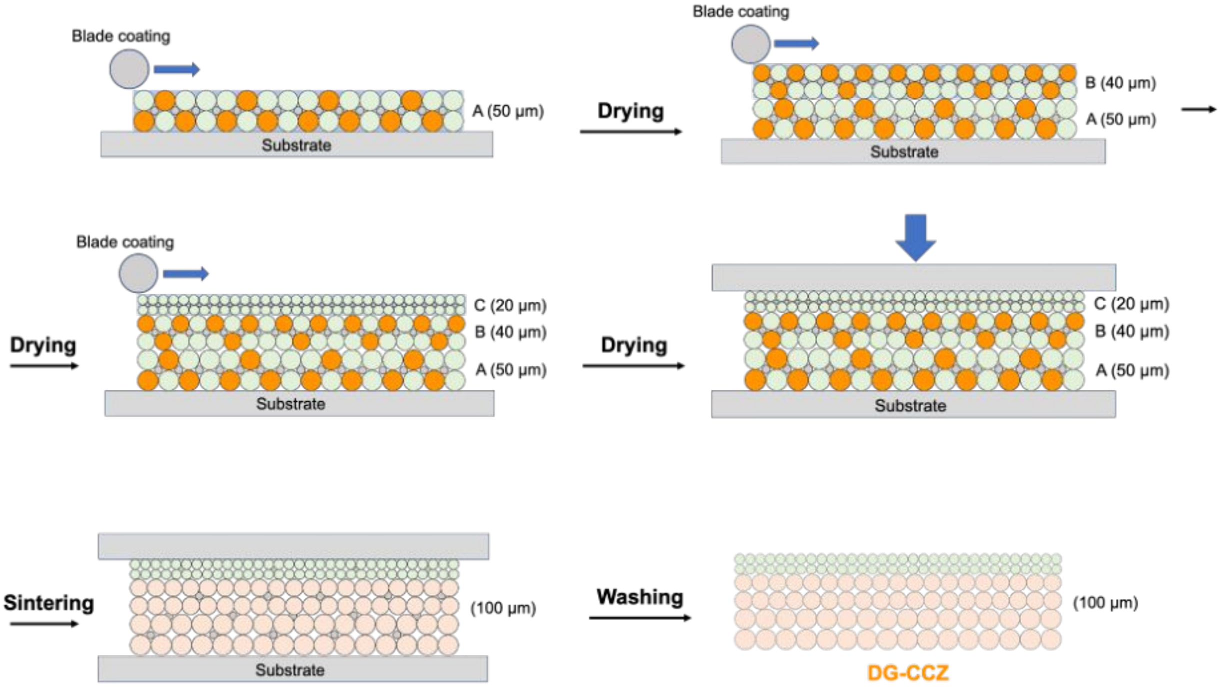

Figure S2. Schematic diagram of preparation process of DG-CCZ current collectors.

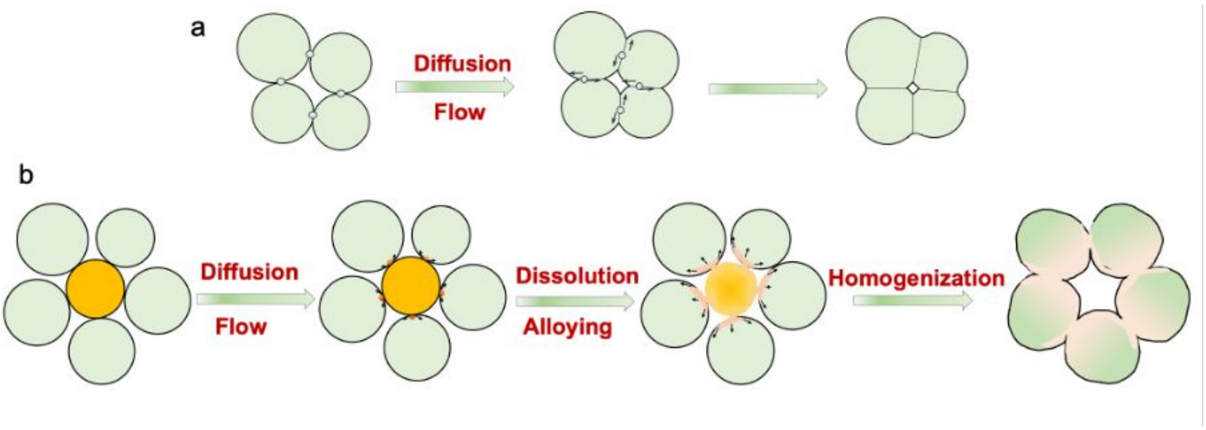

Figure S3. Model diagram of the powder sintering process. Schematic diagram of the sintering preparation process of (a) single-component metal powder and (b) dual-component metal powder.

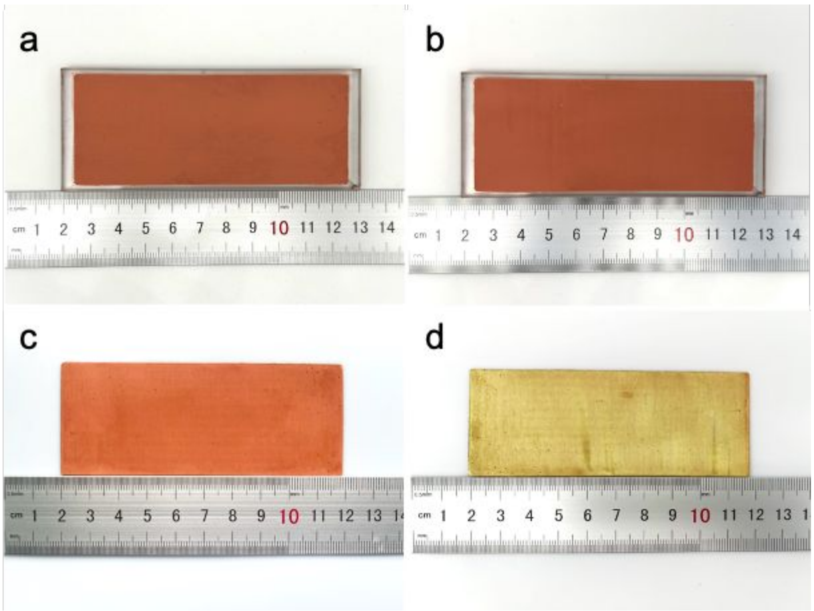

Figure S4. Digital photographs of the prepared DG-CCZ samples with the front (a) and back (b) sides before sintering. Digital photographs of the prepared DG-CCZ samples with the front (c) and back (d) sides after sintering.

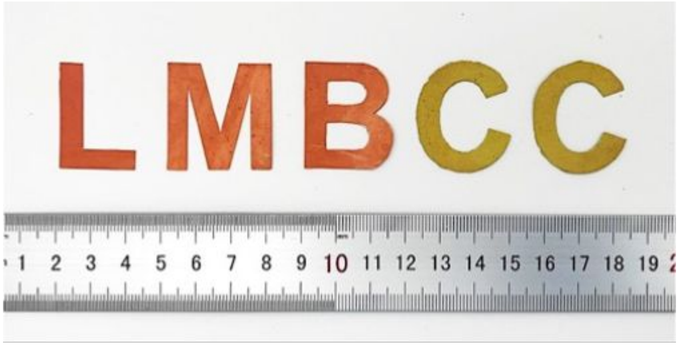

Figure S5. Digital photograph of the as-prepared DG-CCZ current collectors with different shapes.

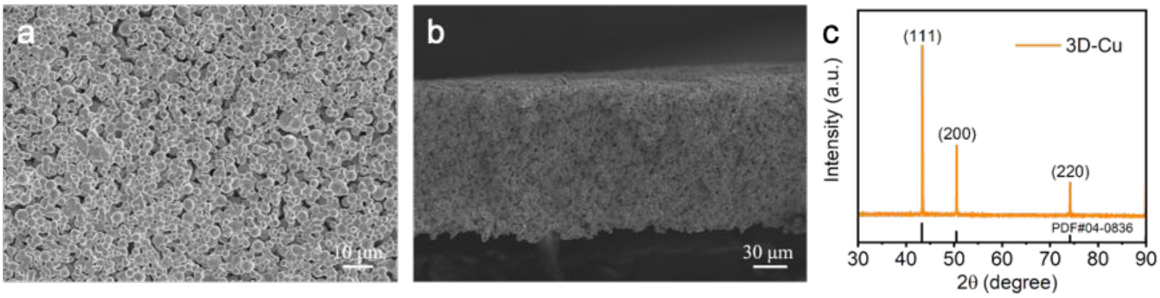

Figure S6. (a) Top-view and (b) sectional-view SEM images of the 3D Cu current collector. (c) XRD patterns of 3D Cu current collector.

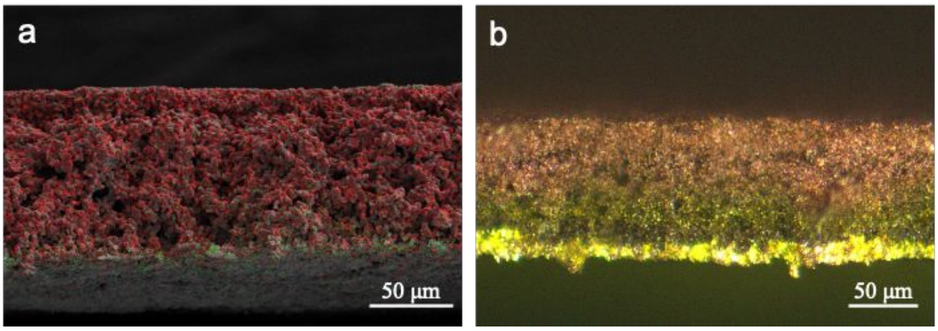

Figure S7. (a) Cross-sectional EDS mapping image (a) and optical microscopy images (b) of DG-CCZ current collector.

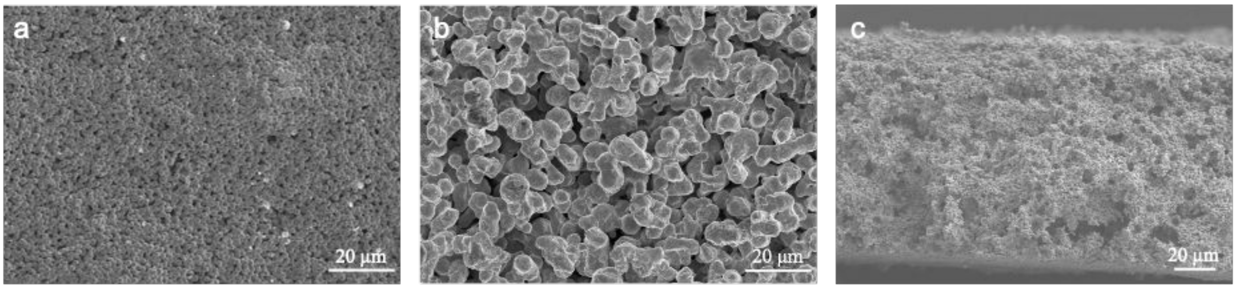

Figure S8. (a) Top-view, (b) bottom-view, and (c) sectional-view SEM images of DG-CCZ current collector (sintering at 350 °C).

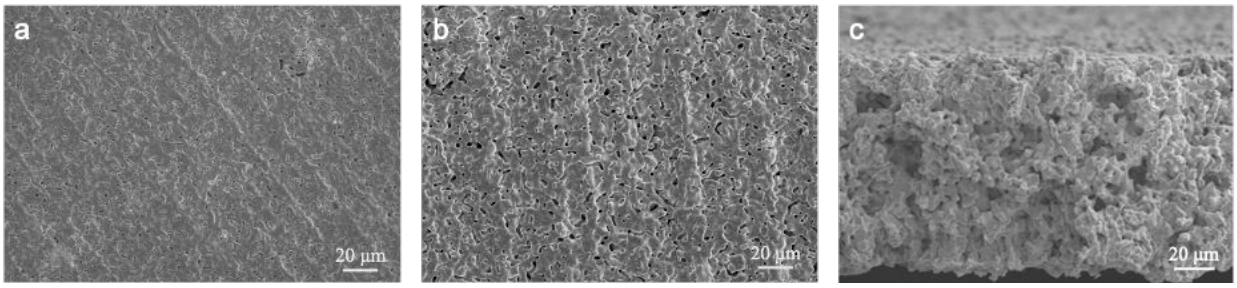

Figure S9. (a) Top-view, (b) bottom-view, and (c) sectional-view SEM images of DG-CCZ current collector. (sintering at 550 °C).

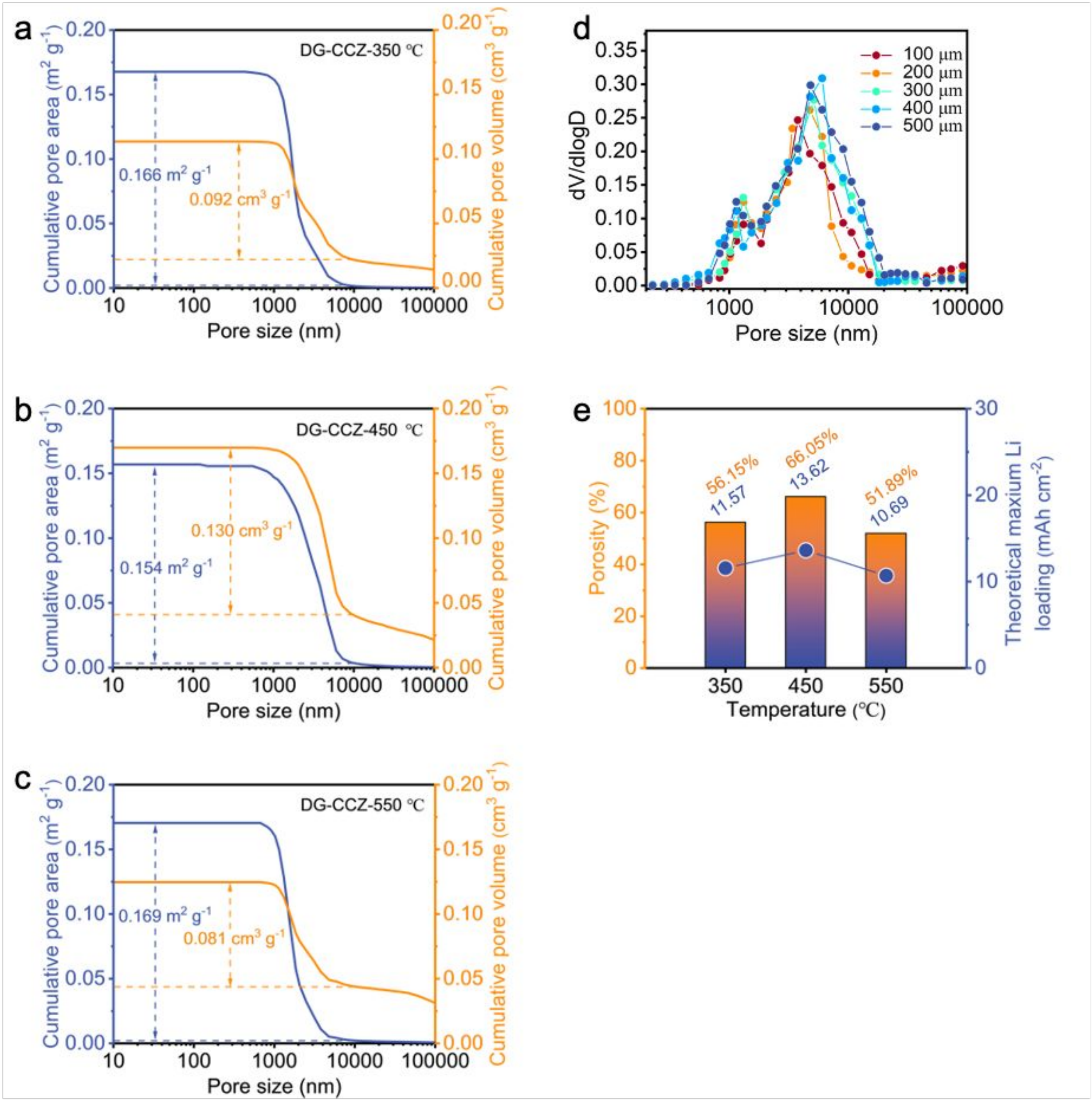

Figure S10. Porosity analysis for DG-CCZ current collectors by mercury porosimetry. Cumulative pore volume and cumulative pore area of DG-CCZ current collector sintered at (a) 350 ℃, (b) 450 ℃, (c) 550 ℃. (d) Pore size distribution of DG-CCZ sintered at 450 ℃ with thickness range from 100-500 μm. (e) The porosity of the as-prepared DG-CCZ current collector (100 μm) at different sintering temperatures and the corresponding maximum theoretical Li metal loading.

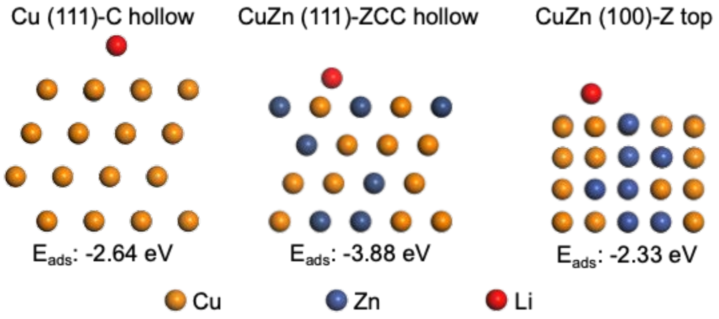

Figure S11. The adsorption energy of a Li atom on Cu (111), CuZn (111), and CuZn (100) surface.

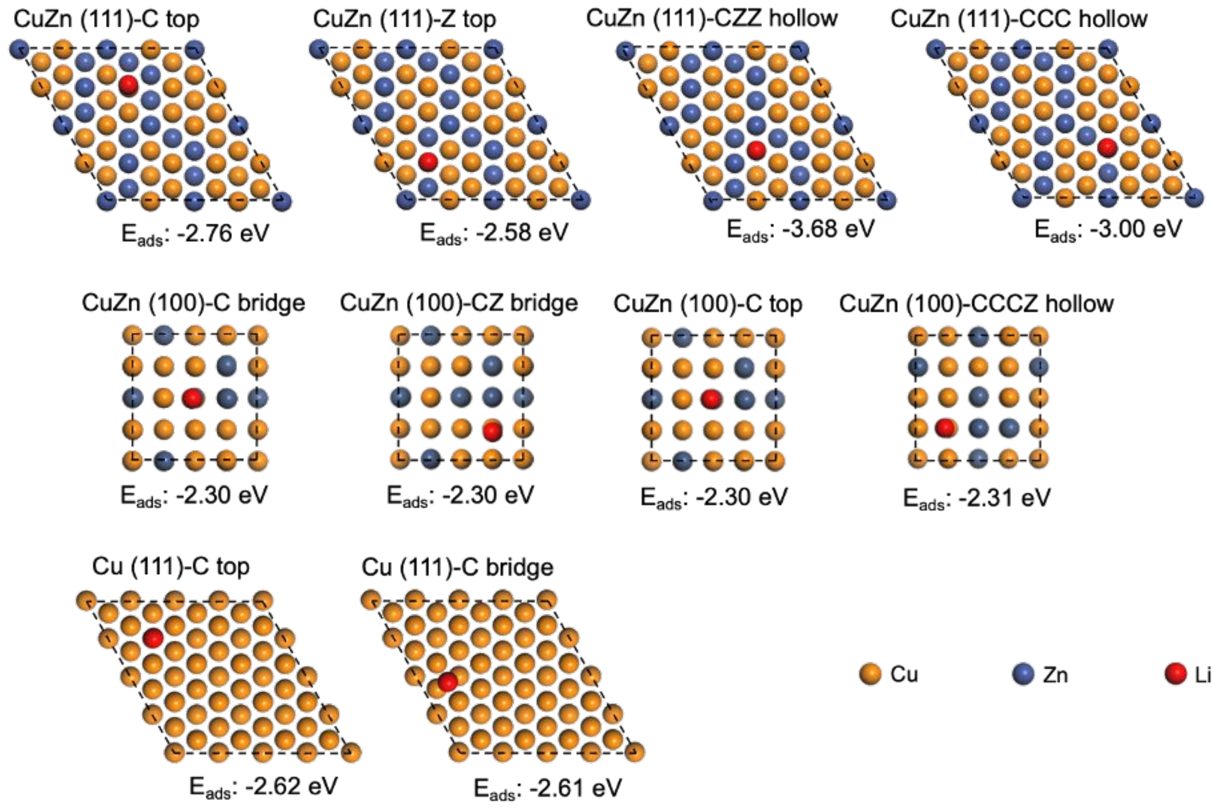

Figure S12. The adsorption energy of a Li atom on other sites of CuZn (111), CuZn (100) and Cu (111) surface.

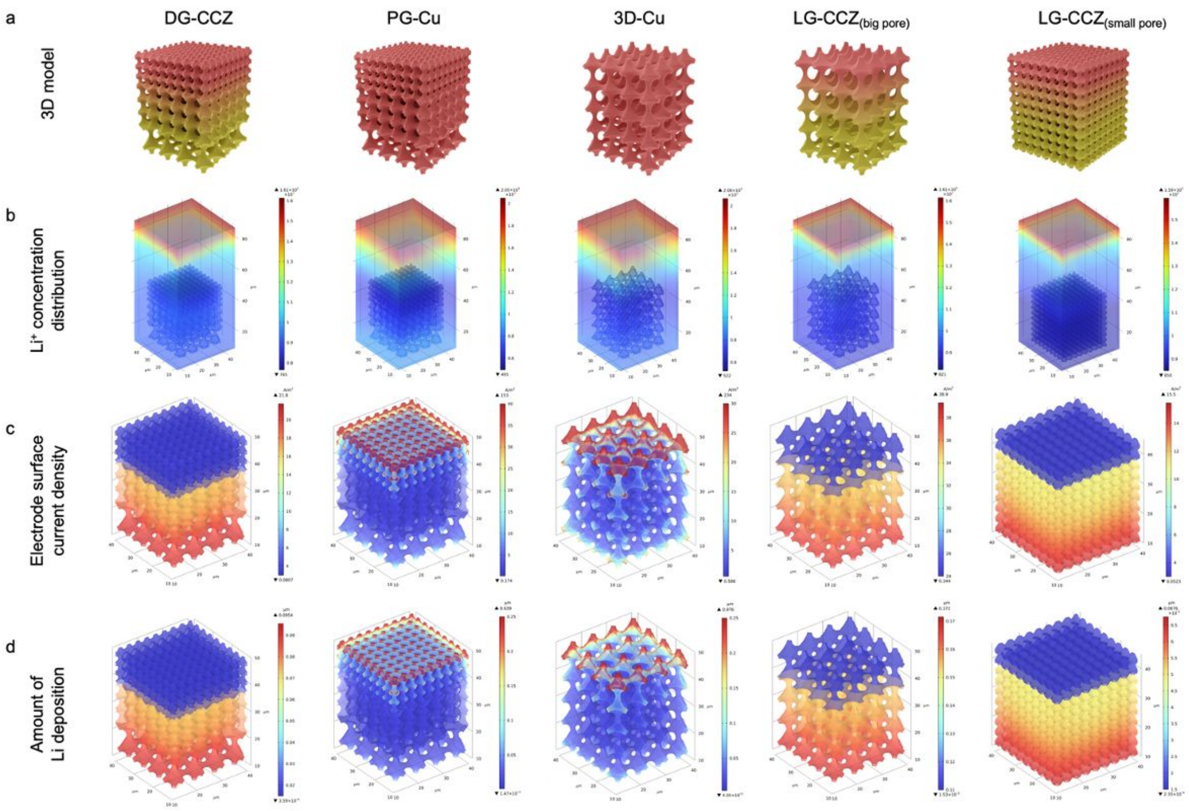

Figure S13. COMSOL simulation results of DG-CCZ, pore gradient Cu (PG-Cu), 3D Cu, LG-CCZ$_{(big\ pore)}$ and LG-CCZ$_{(small\ pore)}$-based half cell. (a) The schematic diagram of 3D models for the designed DG-CCZ, PG-Cu, 3D Cu, LG-CCZ$_{(big\ pore)}$ and LG-CCZ$_{(small\ pore)}$. (b) COMSOL simulation results of Li-ion concentration distribution in different models. (c) COMSOL simulation results of current density in different electrode surfaces. (d) COMSOL simulation results of the amount of Li deposition in different electrode surfaces.

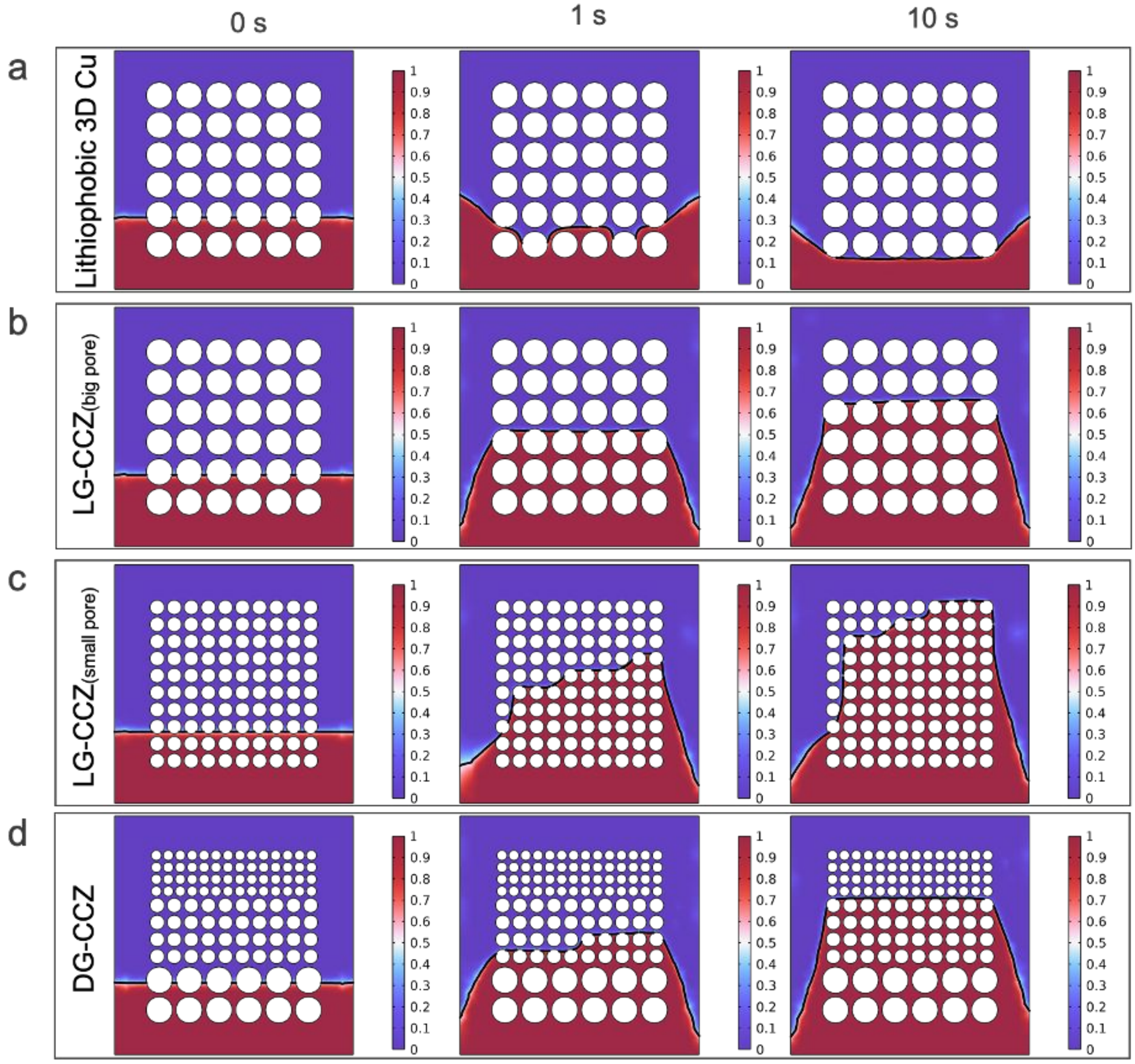

Figure S14. Simulation results of molten Li infusion behavior in (a) lithiophobic 3D Cu, (b) LG-$CCZ_{(big\ pore)}$, (c) LG-$CCZ_{(small\ pore)}$, and (d) DG-CCZ current collector.

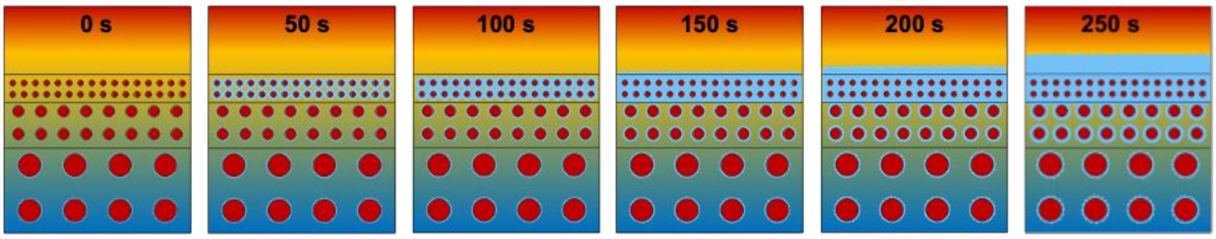

Figure S15. Simulation results of electrochemical deposition behavior of Li in PG-Cu current collectors.

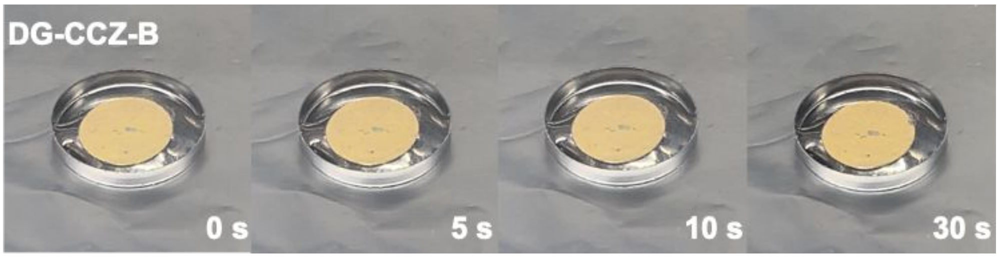

Figure S16. Dynamic wetting process of molten Li in inverted DG-CCZ current collectors.

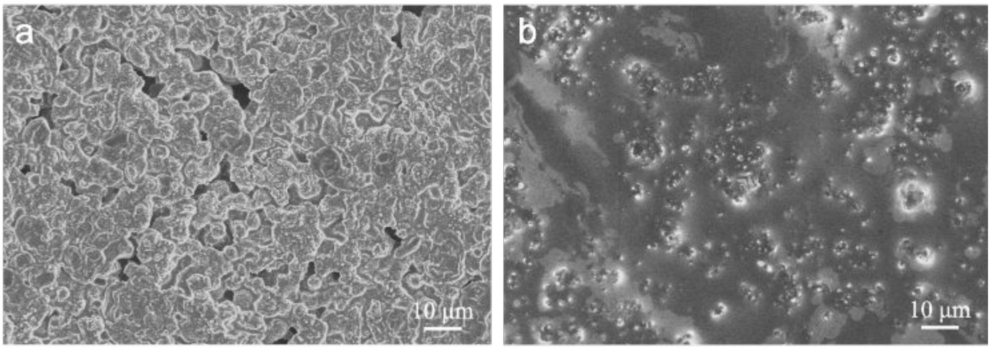

Figure S17. Top-view (a) and bottom-view (b) SEM images of Li/ DG-CCZ after plating 10 mAh cm^{-2}.

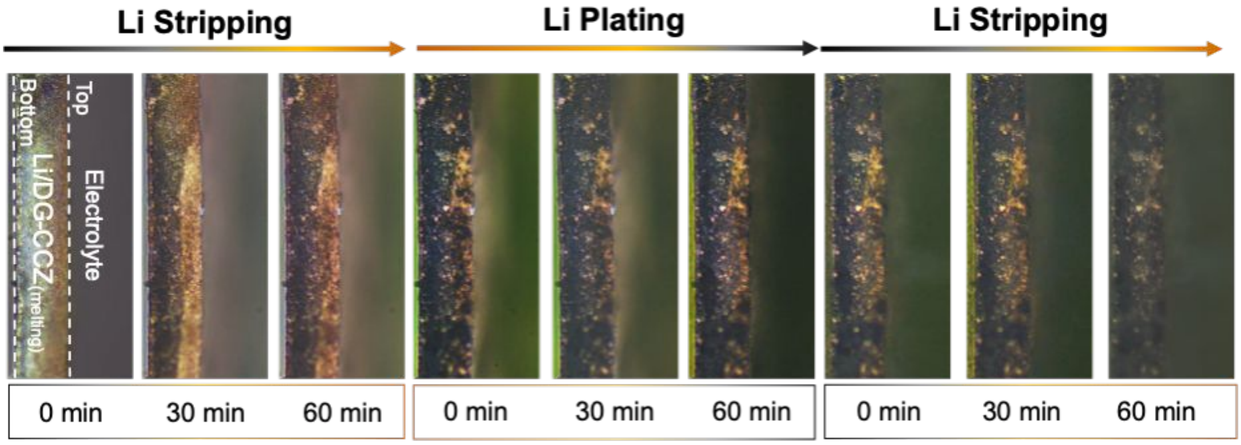

Figure S18. Dynamic electrochemical behavior of metallic Li dissolution and deposition in Li/DG-CCZ$_{(melting)}$ captured by in-situ optical electron microscopy.

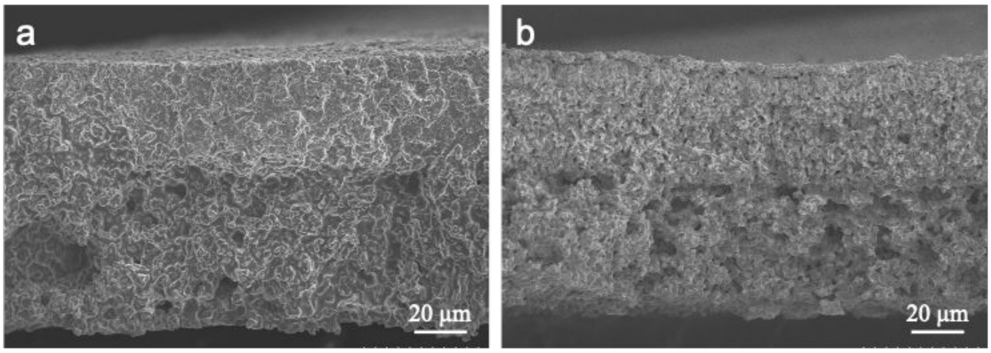

Figure S19. Cross-sectional SEM images of cycled Li/DG-CCZ (in-situ optical cell) anodes under a plating capacity of (a) 10 mAh cm^{-2} and (b) fully stripped.

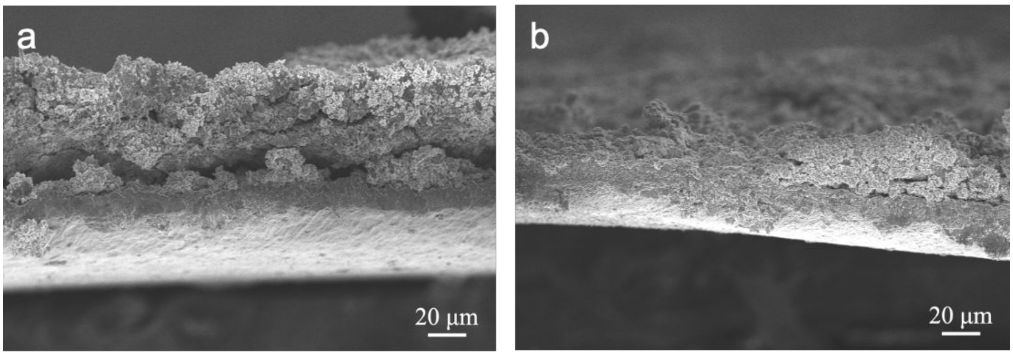

Figure S20. Cross-sectional SEM images of cycled Li/PL-Cu (in-situ optical cell) anodes under a plating capacity of (a) 10 mAh cm^{-2} and (b) fully stripped.

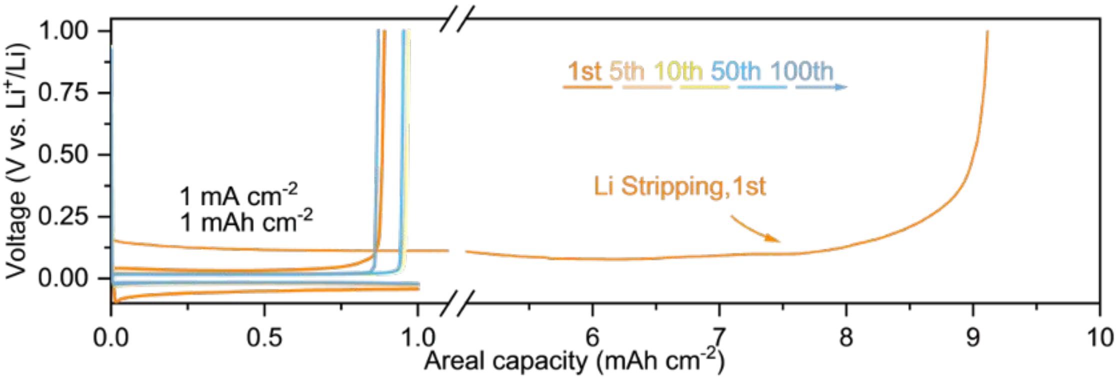

Figure S21. Charge-discharge voltage profiles of Li/PL-Cu composite electrode.

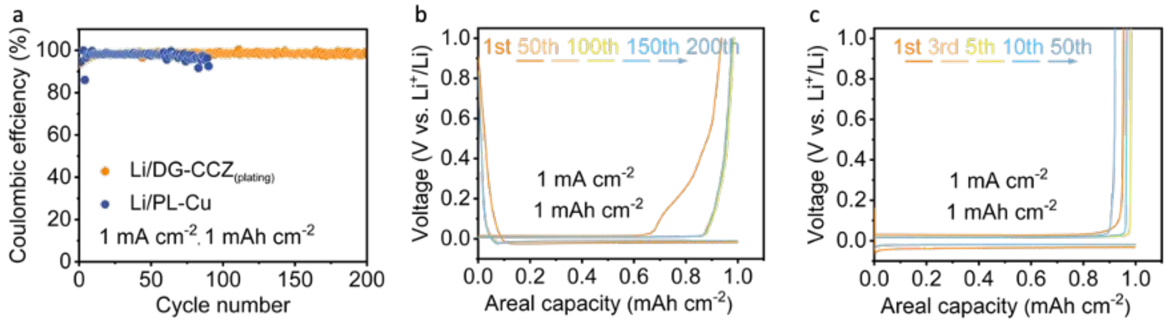

Figure S22. (a) Coulombic efficiencies of Li plating/stripping on the DG-CCZ current collectors in half cells with a plating/stripping capacity of 1 mAh cm^{-2} under 1 mA cm^{-2}. Voltage profiles of Li plating/stripping on the (b) DG-CCZ and (c) PL-Cu current collectors in half cells with a plating/stripping capacity of 1 mAh cm^{-2} under 1 mA cm^{-2}.

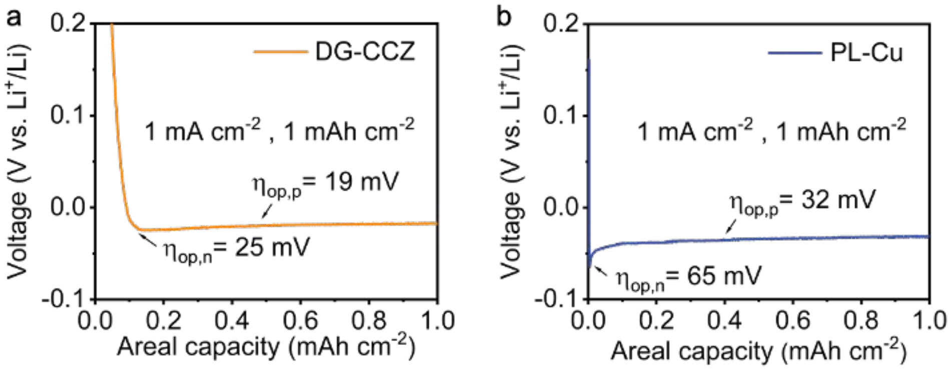

Figure S23. Voltage-time curves of (a) DG-CCZ and (b) PL-Cu during Li metal nucleation and deposition with a plating/stripping capacity of 1 mAh cm^{-2} under 1 mA cm^{-2}.

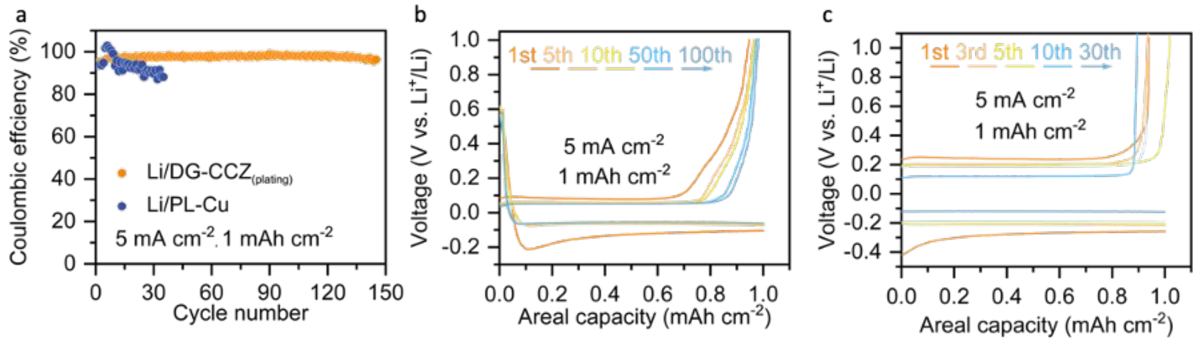

Figure S24. (a) Coulombic efficiencies of Li plating/stripping on the DG-CCZ current collectors in half cells with a plating/stripping capacity of 1 mAh cm^{-2} under 5 mA cm^{-2}. Voltage profiles of Li plating/stripping on the (b) DG-CCZ and (c) PL-Cu current collectors in half cells with a plating/stripping capacity of 1 mAh cm^{-2} under 5 mA cm^{-2}.

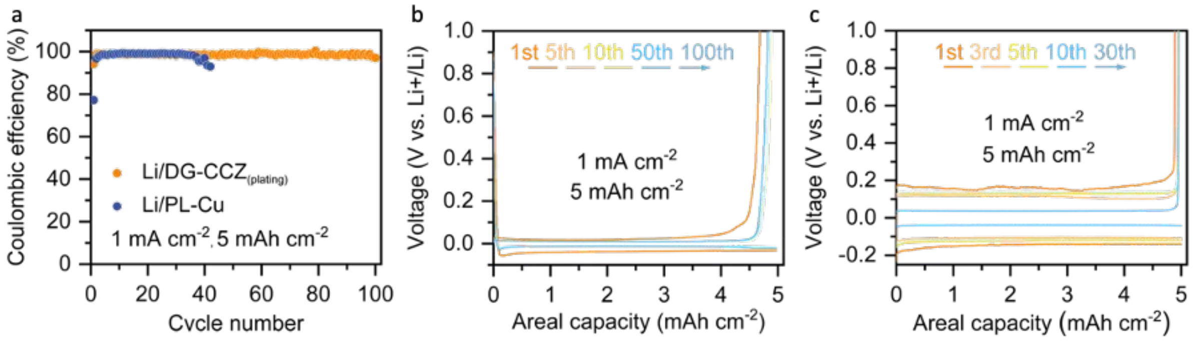

Figure S25. (a) Coulombic efficiencies of Li plating/stripping on the DG-CCZ current collectors in half cells with a plating/stripping capacity of 5 mAh cm^{-2} under 1 mA cm^{-2}. Voltage profiles of Li plating/stripping on the (b) DG-CCZ and (c) PL-Cu current collectors in half cells with a plating/stripping capacity of 5 mAh cm^{-2} under 1 mA cm^{-2}.

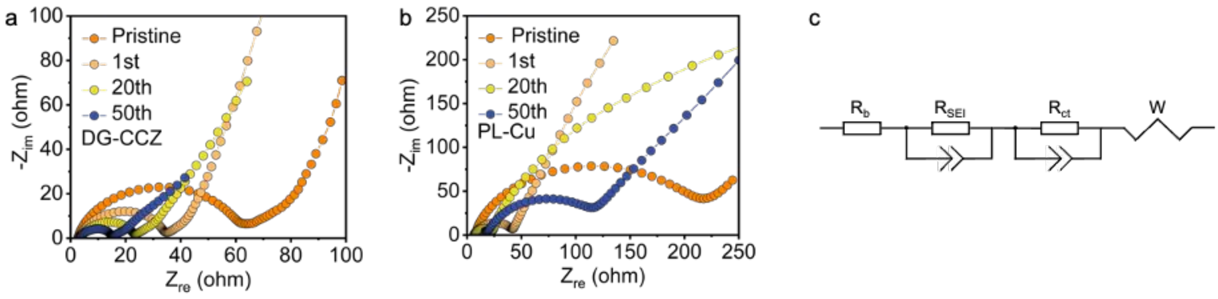

Figure S26. Electrochemical impedance spectroscopy plots of (a) DG-CCZ and (b) PL-Cu half cells after certain cycles under typical current density (1 mA cm^{-2}, 1 mAh cm^{-2}). (c) Equivalent circuit diagram of electrochemical impedance spectroscopy plots in (a-b).

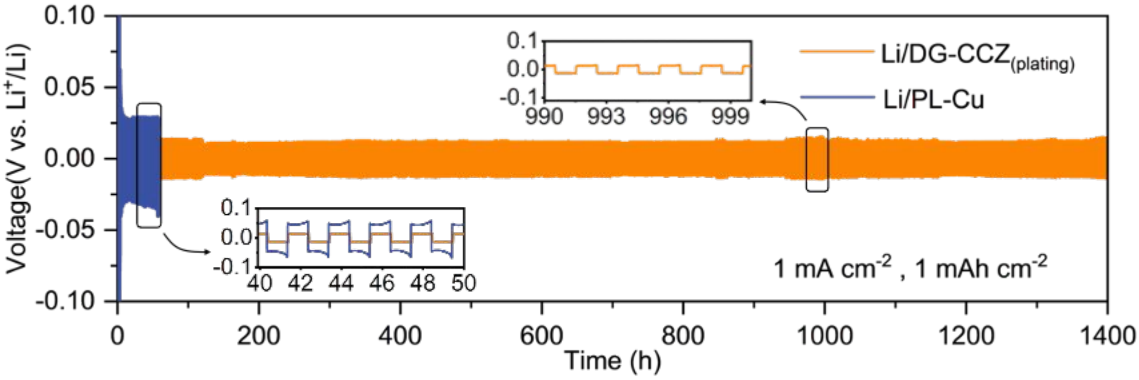

Figure S27. Voltage-time profiles of Li/DG-$CCZ_{(plating)}$ composite electrode in symmetric cells with a plating/stripping capacity of 1 mAh cm^{-2} under 1 mA cm^{-2}.

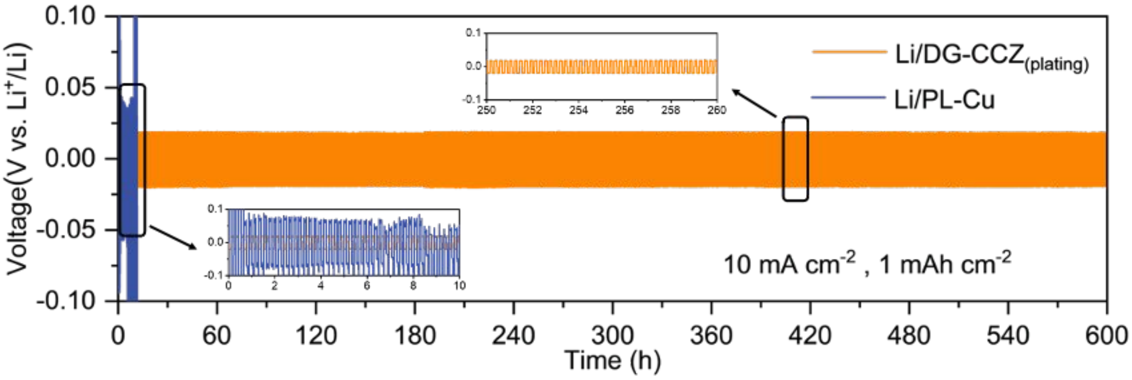

Figure S28. Voltage-time profiles of Li/DG-CCZ$_{(plating)}$ composite electrode in symmetric cells with a plating/stripping capacity of 1 mAh cm^{-2} under 10 mA cm^{-2}.

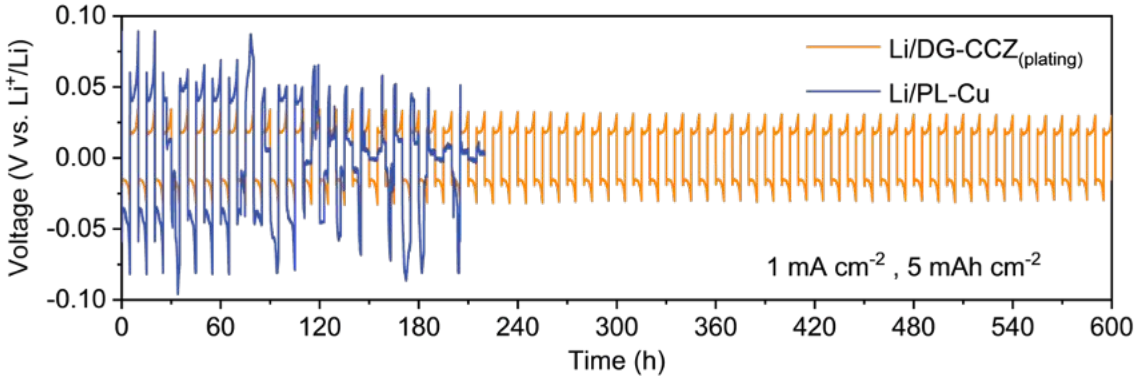

Figure S29. Voltage-time profiles of Li/DG-CCZ$_{(plating)}$ composite electrode in symmetric cells with a current density of 1 mA cm^{-2} under a plating/stripping capacity of 5 mAh cm^{-2}.

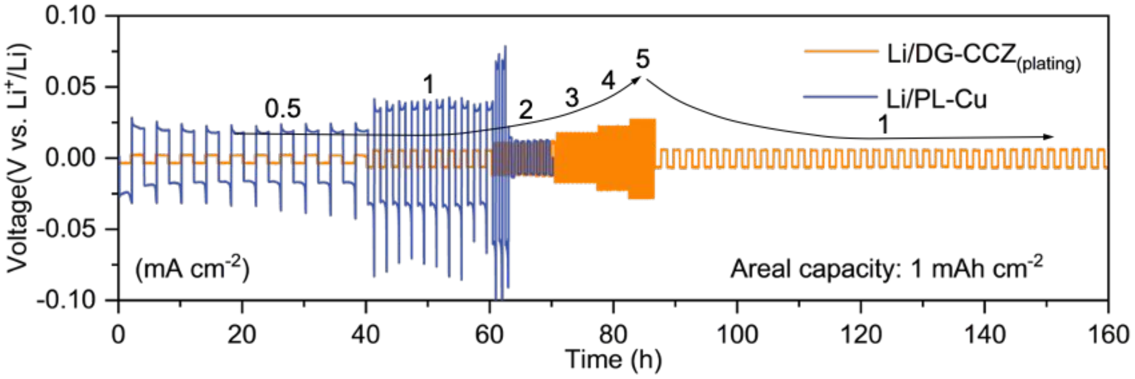

Figure S30. Voltage-time profiles of Li/DG-CCZ$_{(plating)}$ composite electrode symmetric cells under various current densities and cyclic capacity of 1 mAh cm^{-2}.

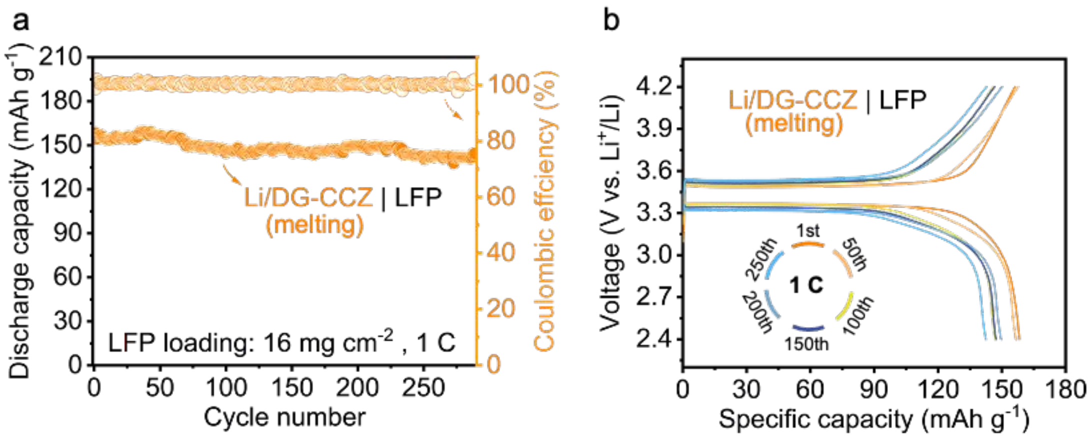

Figure S31. (a) Long-term electrochemical performance of Li/DG-$CCZ_{(melting)}$ | LFP and Li/PL-Cu | LFP full cells at 1 C. (b) Charge-discharge voltage profiles of Li/DG-$CCZ_{(melting)}$ |LFP full cell at 1 C.

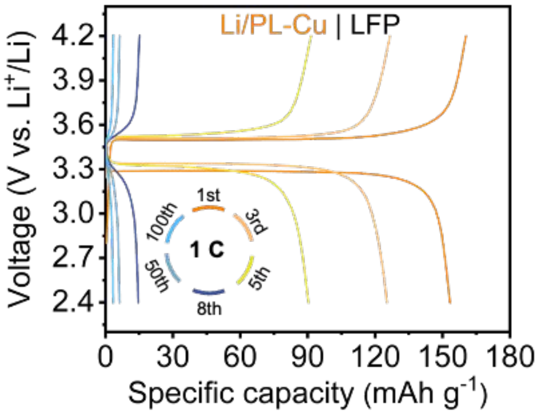

Figure S32. Charge-discharge voltage profiles of Li/PL-Cu | LFP full cell at 1 C.

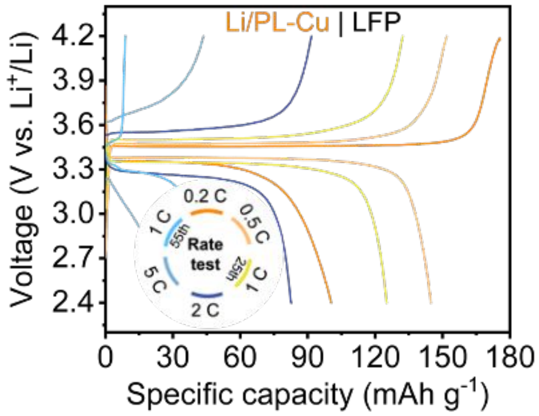

Figure S33. Charge-discharge voltage profiles of Li/PL-Cu | LFP full cell under different rates.

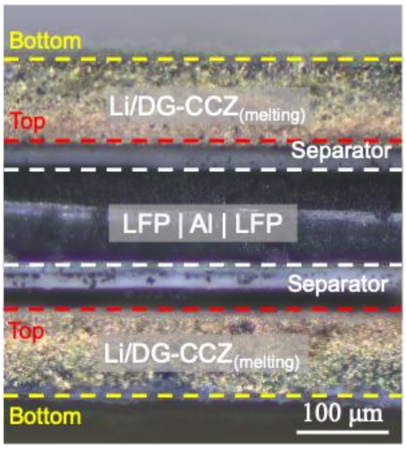

Figure S34. Cross-sectional optical microscope images of pouch cell with a double-coated $LiFePO_4$ cathode and double Li/DG-$CCZ_{(melting)}$ composite anodes.

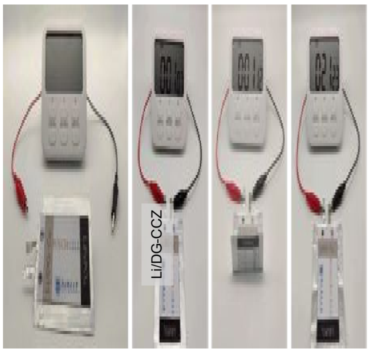

Figure S35. Digital photographs of the timer powered by the Li/DG-$CCZ_{(melting)}$ | LFP | Li/DG-$CCZ_{(melting)}$ pouch cell.

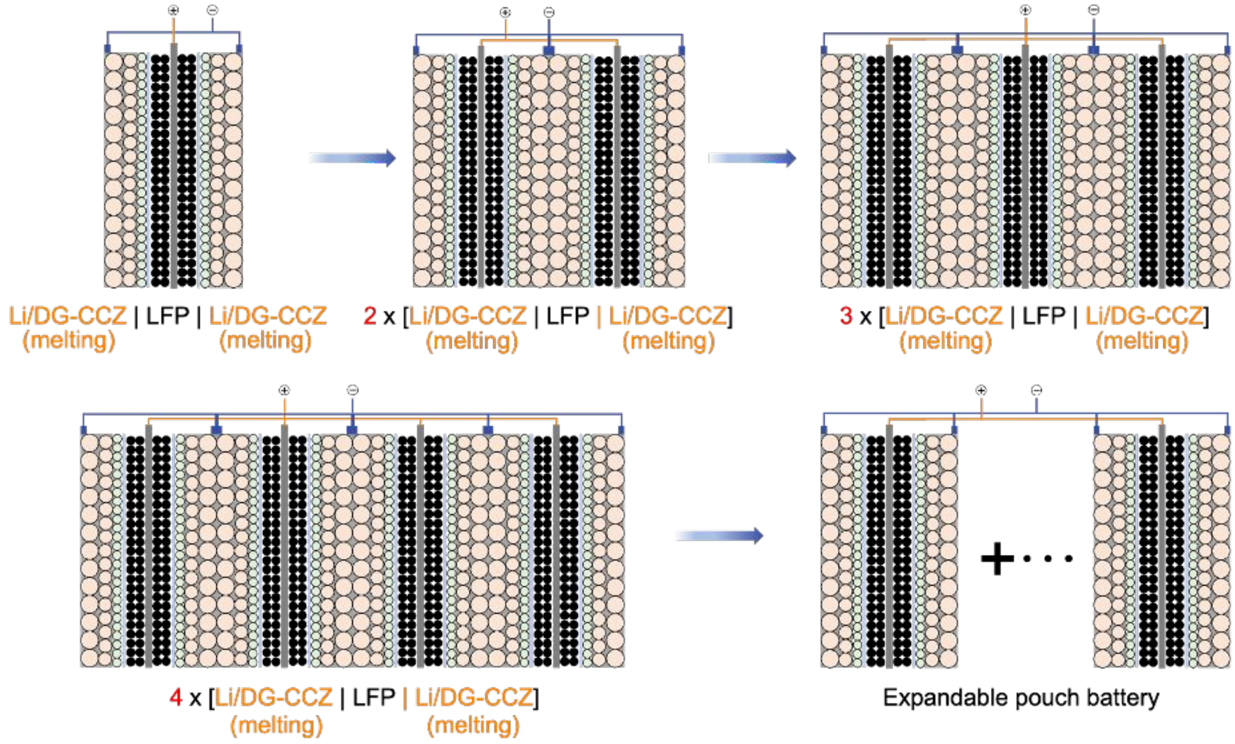

Figure S36. Schematic diagram of pouch cell configuration based on a single layer, double layers, and multiple layers.

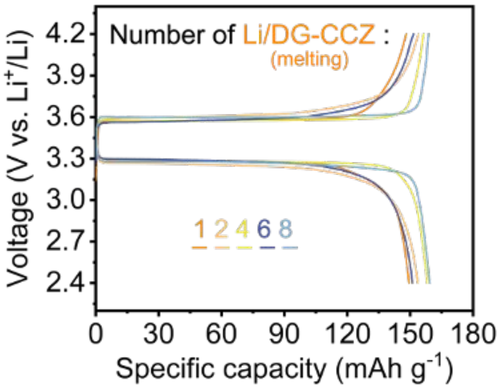

Figure S37. Charge-discharge voltage profiles of Li/DG-CCZ$_{(melting)}$ | LFP | Li/DG-CCZ$_{(melting)}$ pouch cell with different electrode configurations; the number inset of the figure refers to the number of Li/DG-CCZ$_{(melting)}$ anodes.

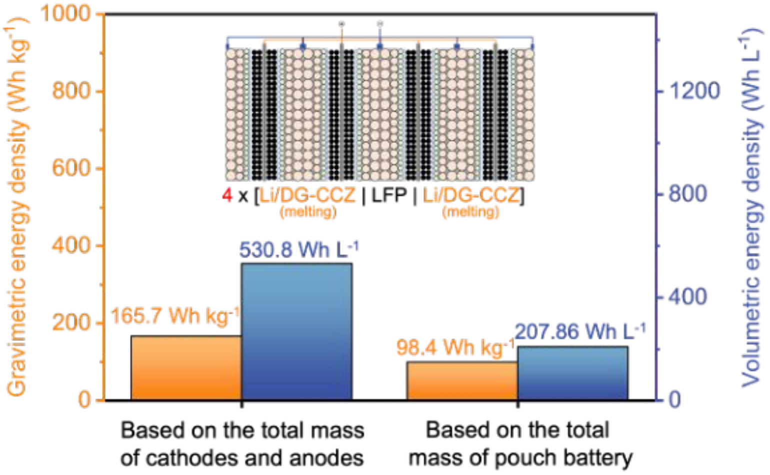

Figure S38. The bar chart demonstrates the calculated gravimetric/volumetric energy densities of pouch cells based on Li/DG-$CCZ_{(melting)}$ | LFP| Li/DG-$CCZ_{(melting)}$ configuration; the inset is a schematic depiction of a multiple electrode stacking cell.

Table S1. Electrochemical impedance fitted parameters by an equivalent circuit model (Figure S26c) for Figure S26a, b.

	**DG-CCZ**			**PL-Cu**		
	R_b (Ω)	R_{SEI} (Ω)	R_{ct} (Ω)	R_b (Ω)	R_{SEI} (Ω)	R_{ct} (Ω)
Pristine	2.71	56.64	46.02	7.15	201.9	997.8
1st cycle	3.26	30.33	23.05	47.01	35.81	607.5
20th cycle	3.78	18.67	21.52	6.20	16.73	316.5
50th cycle	3.33	11.43	7.96	16.76	107.7	120.3

Table S2. Parameters of Li/DG-CCZ$_{(melting)}$ | LFP | Li/DG-CCZ$_{(melting)}$ pouch cell.

Physical parameters of each component of Li/DG-CCZ$_{(melting)}$ | LFP | Li/DG-CCZ$_{(melting)}$ pouch cell

	Cathodes	Anodes	Separator	Electrolyte	Lugs	Plastic films	Total
Mass (mg)	142.03	269.26	20.00	100.00	64.31	448 .76	1045.02
Mass ratio (%)	13.59	25.83	1.92	9.57	6.15	42.94	100

Electrochemical performance parameters of Li/DG-CCZ$_{(melting)}$ | LFP | Li/DG-CCZ$_{(melting)}$ pouch cell (Based on the total mass of the cathode and anode)

Cycles	1st	3rd	5th	10th	37th	50th	100th
Discharge voltage (V)	3.27	3.27	3.26	3.25	3.25	3.19	3.12
Discharge capacity (mAh g^{-1})	145.2	148.6	150.3	153.6	154.4	159.5	145.2
Gravimetric energy density (Wh kg^{-1})	147.9	151.3	152.6	155.5	159.4	158.5	141.1
Volumetric energy density (Wh L^{-1})	506.6	518.3	522.6	532.5	545.8	542.7	483.2

Table S3. Electrochemical performance parameters of different number of anodes based pouch cell.

Electrochemical performance parameters of different numbers of anode pouch cells at the 10th cycling (Based on the total mass of the cathode and anode)

Number of anodes	1	2	4	6	8
Discharge voltage (V)	3.28	3.25	3.27	3.29	3.28
Discharge capacity (mAh g^{-1})	149.5	153.6	158.2	151.0	159.3
Gravimetric energy density (Wh kg^{-1})	150.9	155.5	163.9	156.4	165.7
Volumetric energy density (Wh L^{-1})	490.4	532.5	494.2	472.2	530.8

Table S4. Comparison of electrochemical performances of different Li metal composite anodes.

Composite anode types	Methods	Component of pouch cell	Pouch-cell performance (current rate/C, mA g^{-1}, cycle number/n, capacity retention)	Number of anodes	Ref
Li/3D-CPA	Electrophoretic deposition	NCM | Li/3D-CPA	0.5 C, 100, 87.2%	1	5
Li/E-Cu	Electrophoretic deposition	NCM | Li/E-Cu	0.2 C, 50, 84.0%	1	6
3D Mg_xLi_y/LiF-Li-rGO	Thermal molten	NCM | 3D Mg_xLi_y/LiF-Li-rGO	0.2 C/0.4 C, 150, 85.0%	6	7
Li/G@PVDF-HFP	One-step thermal fluorination method	NCM | Li/G@PVDFHFP	0.1 C, 140, 85%	N/A	8

Li-Mg-Ca	Molten, folding and rolling	NCM | Li-Mg-Ca	N/A, 20, 80.0%	N/A	9
Li/CF	One-step rolling	S | Li/CF	0.1 C, 100, 98.0%	1	10
Housed Li	Rolling	LFP | Housed Li	0.5 C, 80, 85.0%	1	11
G@Li	Slurry coating	NCM | G@Li	0.1 C/0.3 C, 470, 76%	1	12
Li/DICu	Electrophoretic deposition	S@porous C | Li/DICu	0.5 C, 100, 65.5%	1	13
Li/Cu@Cu_xO	Electrophoretic deposition	LFP | Li/Cu@Cu_xO	1 C, 380, 96.8%	1	14
Li/UP-Cu-50	Electrophoretic deposition	LFP | Li/UP-Cu-50	1 C, 120, 99.1%	1	15
Li/IBP-Cu	Electrophoretic deposition	LFP | Li/IBP-Cu | LFP	1 C, 100, 86.8%	1	16
Li/Au@PHCF	Electrophoretic deposition	NCM | Li/Au@PHCF	0.5 C, 160, 86.9%	1	17
Li@G	Thermally initiate conversion	NCM | Li@G	0.2 C, 75, 80.0%	1	18
PGNLi	Electrophoretic deposition	PV_2O_5 | PGNLi | PV_2O_5	N/A, 47, 67.1%	1	19
SR-G-Li	Spray-painting	LFP | SR-G-Li	1 C, 200, N/A	1	20
LSIS@Li	Via solvent-free dry wiping process	LCO | LSIS@Li	0.3 C/0.5 C, 145, 80%	4	21
PANI&MF- Li	Electrophoretic deposition	NCM | PANI&MF- Li	0.2 C, 80, 85.0%	1	22
Li-Zn	Melted and rolling	NCM | Li-Zn	50 mA, 50, 99.1%	1	23
Li/DG-CCZ	Thermal molten	Li/DG-CCZ | LFP | Li/DG-CCZ	1 C, 110, 91.7%	8	This work

REFERENCES

(1) Peng, G.; Zheng, Q.; Luo, G.; Zheng, D.; Feng, S.-P.; Khan, U.; Akbar, A. R.; Luo, H.; Liu, F. A Gradient Lithiophilic Structure for Stable Lithium Metal Anodes with Ultrahigh Rate and Ultradeep Capacity. *Small.* **2023**, *19*, 2303787.

(2) Giannozzi, P.; Baroni, S.; Bonini, N.; Calandra, M.; Car, R.; Cavazzoni, C.; Ceresoli, D.; Chiarotti, G. L.; Cococcioni, M.; Dabo, I.; et al. QUANTUM ESPRESSO: A Modular and Open-Source Software Project for Quantum Simulations of Materials. *J. Phys.: Condens. Matter*. **2009**, *21*, 395502.

(3) Perdew, J. P.; Burke, K.; Ernzerhof, M. Generalized Gradient Approximation Made Simple. *Phys. Rev. Lett.* **1996**, *77*, 3865-3868.

(4) Grimme, S.; Ehrlich, S.; Goerigk, L. Effect of the Damping Function in Dispersion Corrected Density Functional Theory. *J. Comput. Chem.* **2011**, *32*, 1456-1465.

(5) Noh, H.-J.; Lee, M.-H.; Kim, B. G.; Park, J.-H.; Lee, S.-M.; Choi, J.-H. 3D Carbon-Based Porous Anode with a Pore-Size Gradient for High-Performance Lithium Metal Batteries. *ACS Appl. Mater. Interfaces.* **2021**, *13*, 55227–55234.

(6) Lin, L.; Suo, L.; Hu, Y.; Li, H.; Huang, X.; Chen, L. Epitaxial Induced Plating Current-Collector Lasting Lifespan of Anode-Free Lithium Metal Battery. *Adv. Energy Mater.* **2021**, *11* , 2003709.
(7) Xu, Q.; Yang, X.; Rao, M.; Lin, D.; Yan, K.; Du, R.; Xu, J.; Zhang, Y.; Ye, D.; Yang, S.; Zhou, G.; Lu, Y.; Qiu, Y. High Energy Density Lithium Metal Batteries Enabled by a Porous Graphene/MgF_2 Framework. *Energy Storage Mater.* **2020**, *26*, 73-82.
(8) Liu, Z.; He, B.; Zhang, Z.; Deng, W.; Dong, D.; Xia, S.; Zhou, X.; Liu, Z. Lithium/Graphene Composite Anode with 3D Structural LiF Protection Layer for High-Performance Lithium Metal Batteries. *ACS Appl. Mater. Interfaces.* **2022**, *14*, 2871-2880.
(9) Deng, Y.; Gao, J.; Wang, M.; Luo, C.; Zhou, C.; Wu, M. Homogenizing the Li-Ion Flux by Multi-Element Alloying Modified for 3D Dendrite-Free Lithium Anode. *Energy Storage Mater.* **2022**, *48*, 114-122.
(10) Shi, P.; Li, T.; Zhang, R.; Shen, X.; Cheng, X.-B.; Xu, R.; Huang, J.-Q.; Chen, X.-R.; Liu, H.; Zhang, Q. Lithiophilic LiC_6 Layers on Carbon Hosts Enabling Stable Li Metal Anode in Working Batteries. *Adv. Mater.* **2019**, *31*, 1807131.
(11) Shen, X.; Cheng, X.; Shi, P.; Huang, J.; Zhang, X.; Yan, C.; Li, T.; Zhang, Q. Lithium–Matrix Composite Anode Protected by a Solid Electrolyte Layer for Stable Lithium Metal Batteries. *J. Energy Chem.* **2019**, *37*, 29-34.
(12) Yu, Y.; Ying, D.; Xu, S.; Guo, Q.; Li, Y.; Wang, S.; Zhou, X.; Shao, G.; Liu, Z. Graphene Coated Lithium Foil Anode Enables Long Cycle Life Li Metal Pouch Cells. *Carbon.* **2023**, *215*, 118498.
(13) Chen, J.; Zhao, J.; Lei, L.; Li, P.; Chen, J.; Zhang, Y.; Wang, Y.; Ma, Y.; Wang, D. Dynamic Intelligent Cu Current Collectors for Ultrastable Lithium Metal Anodes. *Nano Lett.* **2020**, *20*, 3403-3410.
(14) Chen, J.; Qiao, X.; Fu, W.; Han, X.; Wu, Q.; Wang, Y.; Zhang, Y.; Shi, L.; Zhao, J.; Ma, Y. Lithiophilic Hyperbranched Cu Nanostructure for Stable Li Metal Anodes. *SmartMat.* **2023**, *4*, e1174.
(15) Li, S.; Chen, J.; Liu, G.; Wu, H.; Chen, H.; Li, M.; Shi, L.; Wang, Y.; Ma, Y.; Zhao, J. Ultralight Porous Cu Nanowire Aerogels as Stable Hosts for High Li-Content Metal Anodes. *ACS Appl. Mater. Interfaces.* **2022**, *14*, 56697-56706.
(16) Chen, J.; Li, S.; Qiao, X.; Wang, Y.; Lei, L.; Lyu, Z.; Zhao, J.; Zhang, Y.; Liu, R.; Liang, Q.; Ma, Y. Integrated Porous Cu Host Induced High-Stable Bidirectional Li Plating/Stripping Behavior for Practical Li Metal Batteries. *Small.* **2022**, *18*, 2105999.
(17) Kang, D. W.; Park, S. S.; Choi, H. J.; Park, J.-H.; Lee, J. H.; Lee, S.-M.; Choi, J.-H.; Moon, J.; Kim, B. G. One-Dimensional Porous Li-Confinable Hosts for High-Rate and Stable Li-Metal

Batteries. *ACS Nano.* **2022**, *16*, 11892-11901.
(18) He, B.; Deng, W.; Han, Q.; Zhu, W.; Hu, Z.; Fang, W.; Zhou, X.; Liu, Z. Scalable Fabrication of a Large-Area Lithium/Graphene Anode towards a Long-Life 350 W h Kg^{-1} Lithium Metal Pouch Cell. *J. Mater. Chem. A.* **2021**, *9*, 25558-25566.
(19) Li, Y.; Ye, S.; Lin, J.; Song, Y.; Wu, X.; Zhang, J.; Shao, C.; Su, Z.; Sun, H.; Seferos, D. S. A Pore-Forming Strategy Toward Porous Carbon-Based Substrates for High Performance Flexible Lithium Metal Full Batteries. *Energy Environ. Mater.* **2023**, *6*, e12368.
(20) Bai, M.; Xie, K.; Yuan, K.; Zhang, K.; Li, N.; Shen, C.; Lai, Y.; Vajtai, R.; Ajayan, P.; Wei, B. A Scalable Approach to Dendrite-Free Lithium Anodes via Spontaneous Reduction of Spray-Coated Graphene Oxide Layers. *Adv. Mater.* **2018**, *30*, 1801213.
(21) Long, K.; Huang, S.; Wang, H.; Jin, Z.; Wang, A.; Wang, Z.; Qing, P.; Liu, Z.; Chen, L.; Mei, L.; Wang, W. High Interfacial Capacitance Enabled Stable Lithium Metal Anode for Practical Lithium Metal Pouch Cells. *Energy Storage Mater.* **2023**, *58*, 142-154.
(22) Hu, Z.; Deng, W.; He, B.; Liang, J.; Zhou, X.; Liu, Z. Self-Adaptive 3D Skeleton with Charge Dissipation Capability for Practical Li Metal Pouch Cells. *Nano Energy.* **2022**, *93*, 106805.
(23) Li, X.; Zhu, R.; Jiang, H.; Yu, Y.; Wan, W.; Li, X.; Wang, C.; Huang, Y. Thickness-Controllable Li–Zn Composite Anode for High-Energy and Low-N/P Ratio Lithium Metal Batteries. *J. Mater. Chem. A.* **2022**, *10*, 11246-11253.
